# Maximum Lyapunov exponent-based multiple chaotic slime mold algorithm for real-world optimization

**DOI:** 10.1038/s41598-023-40080-1

**Published:** 2023-08-07

**Authors:** Jiaru Yang, Yu Zhang, Ting Jin, Zhenyu Lei, Yuki Todo, Shangce Gao

**Affiliations:** 1 Faculty of Engineering, University of Toyama, Toyama-shi, 930-8555 Japan; 2 School of Science, Nanjing Forestry University, Nanjing, 210037 China; 3 Faculty of Electrical, Information and Communication Engineering, Kanazawa University, Ishikawa, 9201192 Japan

**Keywords:** Computational science, Information technology, Software

## Abstract

Slime mold algorithm (SMA) is a nature-inspired algorithm that simulates the biological optimization mechanisms and has achieved great results in various complex stochastic optimization problems. Owing to the simulated biological search principle of slime mold, SMA has a unique advantage in global optimization problem. However, it still suffers from issues of missing the optimal solution or collapsing to local optimum when facing complicated problems. To conquer these drawbacks, we consider adding a novel multi-chaotic local operator to the bio-shock feedback mechanism of SMA to compensate for the lack of exploration of the local solution space with the help of the perturbation nature of the chaotic operator. Based on this, we propose an improved algorithm, namely MCSMA, by investigating how to improve the probabilistic selection of chaotic operators based on the maximum Lyapunov exponent (MLE), an inherent property of chaotic maps. We implement the comparison between MCSMA with other state-of-the-art methods on IEEE Congress on Evolution Computation (CEC) i.e., CEC2017 benchmark test suits and CEC2011 practical problems to demonstrate its potency and perform dendritic neuron model training to test the robustness of MCSMA on classification problems. Finally, the parameters’ sensitivities of MCSMA, the utilization of the solution space, and the effectiveness of the MLE are adequately discussed.

## Introduction

Meta-heuristic strategies are increasingly becoming a widespread way of working out all types of mathematical optimization problems. Unlike the preceded traditional heuristics, meta-heuristics can cope with an extensive and more complex range of problem situations because of their generality, which does not depend on the specific conditions of a particular problem^1,2^. ‘Meta’ can be comprehended as a kind of transcendence and extension of the original object. A meta-heuristic is more of an idea or concept developed on heuristic methods. Strictly speaking, a heuristic is a fixed solution contrived by the characteristics of a given problem to get a better solution. Meta-heuristic is a kind of abstract procedure, that constructs a set of universal process or methodology.

Nowadays, as the computational scale and complexity of various engineering application problems increase, the original traditional optimization algorithms and heuristics may no longer confront the current practical situation^3,4^, e.g., image classification and simulation, building load-bearing structure optimization, solar energy parameter optimization, etc^5^. These problems are multi-dimensional, nonlinear, multi-fitting NP-hard problems^6^, which have posed great challenges to the existing computing system. As a result, computer scientists expect to innovate the whole computing system from hardware and software aspects^7,8^. This is where meta-heuristics arise as an upgrade to algorithms from the underlying architecture. Meta-heuristics are a refinement of heuristics, which are the product of combining stochastic algorithms and local search. They create a process that can get rid of local optimum and carry out a robust search in the solution space by coordinating the interaction between local improvement and operational strategies^9^. During the procedure, search strategies are accustomed to acquire and master the information to find the approximate optimal solution effectively. Therefore, the operating mechanism of the meta-heuristic is not overly dependent on the organizational pattern of a certain situation. This principle can be diffusely applied to the combinatorial optimization and function calculation^10,11^.

In meta-heuristics, swarm intelligence has attracted considerable research interest and attention in the fields of optimization, computational intelligence, and computer science in recent years^12^. It exhibits computational intelligent behavior through simple cooperation between each intelligence and shows much stronger selection ability than an individual in the case of optimal selection^13,14^. Ant colony optimization (ACO) is a cornerstone achievement in the development of systematic swarm intelligence theory. Dorigo et al. investigated the real ant colony route planning and the use of biological pheromone mechanism, using pheromone concentration as a quality index to guide individuals to the shortest path^15^. The next generation population ascertains the superior route throughout the whole space according to the pheromone intensity of the previous generation. The greater the pheromone intensity in a certain route, the more presumably the individuals are to draw in that route. The route with the highest pheromone can be considered as the optimal solution sought by the algorithm^16,17^. ACO has good global search capability and is widely used in many combinatorial optimization areas^18^. For example, Gao et al. improved the k-means clustering idea into ACO and proposed a clustering ant colony algorithm which has got considerable achievements in solving dynamic location routing problems^19^. Particle swarm optimization (PSO) differs from ACO in that PSO pays more attention to the decision-making learning direction and collaborative information sharing when all particles traverse the solution space^20,21^. In per period iteration, per particle is obliged to do a learning judgment on whether to modify the route which is predicated on fitness to measure the global optimal solution and the local optimal solution. Thus, PSO accelerates the convergence rate by extracting the current best, and the particle population has a high convergence rate in terms of exploration. A wide range of PSO-based related study has now been implemented in complex systems, traditional optimization, and even large-scale engineering problems^22^. The above two algorithms are some of the supreme widespread and successful population intelligence algorithms. And then a whole bunch of meta-heuristic algorithms with swarm intelligence ideas emerged, including firefly algorithm^23^, whale optimization algorithm (WOA)^24^, flower pollination algorithm^25^, artificial bee colony algorithm^26^, etc.

Considering the strengths of swarm intelligence, we choose the slime mold algorithm (SMA)^27^ as the underlying algorithm, which is also a biological heuristic algorithm with swarm intelligence proposed recently. SMA is enlightened by the exclusive motor feedback mechanism of slime molds. This algorithm simulates the feedback mimicry of slime bacteria spreading food information, resulting in the exploration of the best pathway to obtain energy. This process considers the adaptive bidirectional feedback of the bio-information waves, allowing the algorithm to strike a counterbalance during the search process. There were also several algorithms for microbial mimicry before. For example,^28^ put forward a slime network founded on an ant colony system to solve the high-dimensional traveler problem. Monismith et al.^29^ draw on the five life forms of biological amoebae to construct an artificial neural network (ANN)-based initial lattice to solve the problems of graph theory and generative networks^30^. Unlike these similarly named bacterial algorithms, SMA primarily uses the adjustment of weights in the feedback to model three different biofeedback morphologies of slime molds. Extensive experimental and algorithmic variant studies demonstrate the robustness and effectiveness of this algorithm in solving optimization problems^31^.

Due to the outstanding performance of SMA in the field of stochastic optimization, numerous exceptional SMA variants have been widely applied to address diverse problems. Houssein et al.^32^ proposed a multi-objective variant of SMA that utilized an information archive to store the Pareto-optimal solutions obtained by individuals in the multi-objective search space. This approach yielded remarkable simulation results on CEC2020 multi-objective benchmark functions and the automotive spring spiral problem. Hu et al.^33^ addressed the issue of induced concentration in the slime molds population by employing a dispersal foraging strategy, effectively maintaining the population diversity. This improved algorithm was successfully applied to feature selection problems in data mining, efficiently identifying optimal information features while maintaining high classification accuracy. In^34^, a hierarchical-guided architecture was introduced to enhance SMA for solving mobile robot path planning problems. Experimental results in multiple environments demonstrated that the path constructed by the hierarchical slime molds population exhibited higher smoothness and faster computation speed. These application examples showcase the unique global stochastic optimization capabilities of SMA. By leveraging the automatic optimization capability and global exploration advantages of slime mold individuals, different improvement strategies are introduced to guide slime molds in performing efficient optimization behaviors, and there is still much ongoing work to explore its relevant properties.

Slime molds dynamically adapt their foraging behavior based on bio-wave feedback on the food. When biofeedback indicates that this area has higher food pheromones, the probability that they will stay in this area and perform a spread search becomes higher. When slime molds engage in this local search behavior, we note that this unique behavior is well aligned with the characteristics of chaotic local search. Among the existing meta-heuristic algorithms, chaotic maps are somewhat generalizable. They can be used extensively in population initialization and in adjusting cross-variance operators to perform an effective local search, thereby increasing the probability of discovering the best solution^35,36^. Therefore, we consider adding chaotic maps to the local search of slime molds, aiming to strengthen the capability of this algorithm in local search and further meliorate the stochasticity and ergodicity of the slime molds search behavior.

At the same time, we focus on the essential property of chaotic maps – the maximum Lyapunov exponent (MLE). This coefficient is a crucial criterion for determining whether a system is performing a chaotic motion. We select the most appropriate chaotic map based on the value of MLE, giving the chance for a second selection of chaotic local operator, and ameliorating the overall capability of the entire algorithm in local exploration. In this study, we creatively put forward an MLE-based multiple chaotic slime mold algorithm (MCSMA). We for the first time select suitable chaotic maps according to their MLE relevance and use a multi-chaotic roulette wheel to incorporate these maps into the local search pattern of slime molds, thus realizing a better balance of exploitation and exploration. To verify the performance of MCSMA, extensive experiments are conducted based on 29 IEEE CEC2017 benchmark function optimization problems, 22 IEEE CEC2011 practical applications, and 7 real-world classification problems. Statistical results show that MCSMA significantly outperforms its peers. Additionally, the parameters’ sensitivities of MCSMA, the utilization of the solution space, and the effectiveness of the MLE are systematically discussed to show more insights into MCSMA.

The remaining part is set up by follows: “Brief description of SMA” section roughly introduces the behavioral pattern and search features of slime molds in the underlying algorithm SMA. In “Multi-chaotic local search operator” section, we describe the nature and features of the chaotic local operator in detail. In “MLE‑based multiple chaotic SMA” section, we explain exhaustively how to adapt the MLE-based weight adjustment mechanism into the chaotic operator. In addition, the whole running process of MCSMA is introduced. The experimental data and results of MCSMA on several test function sets are given and analyzed in “Experimental analysis” section. “Discussion” section conducts a sufficient discussion on the optimal parameters of MCSMA, the movement pattern of the population, and the effectiveness of the MLE-based adjustment mechanism. Finally, we summarize and outline the work done in “Conclusion” section, giving some thoughts on future development.

## Brief description of SMA

Biologists discovered early on that slime molds, as single-celled organisms, exhibit incredible intelligence. Nakagaki et al.^37^ devised an interesting maze experiment in which oats were placed at certain points in the maze, and it was found that the slime molds always chose the path that required the least amount of energy and obtained a sufficient amount of food. Tero et al.^38^ later used slime molds to simulate the railway network throughout the Tokyo area. Experiments showed that in complex combinatorial optimization problems, the network formed by the connection of slime molds approximated the optimal path in engineering. Therefore, the scientists believe that this intelligence of the slime molds can be used in the design of transport networks as well as in complex large-scale simulation experiments.

SMA has thoroughly analyzed the mechanisms of cytoplasmic flow and venous structure change as the slime molds search for food. Slime molds sense food through a tight network of veins, and when the veins sense a food source, a biofeedback wave is propagated by a biological oscillator. When the molds sense this feedback wave, they increase the cytoplasmic concentration in the vein, and the thickness of the vein is positively correlated with the cytoplasmic concentration. The more abundant the food signal, the greater the cytoplasmic concentration and the richer the network of veins leading to food, thus establishing the optimal pathway for foraging. Moreover, when faced with different quality food sources, the slime molds can also rationalize the veins leading to food according to the optimal theory. This algorithm learns the adaptive feedback search strategy and special mechanisms of the slime molds and constructs an efficient mathematical optimization model. SMA includes seek nutrition, wrap up nutrition, and biological oscillator processes.

**Figure 1:**
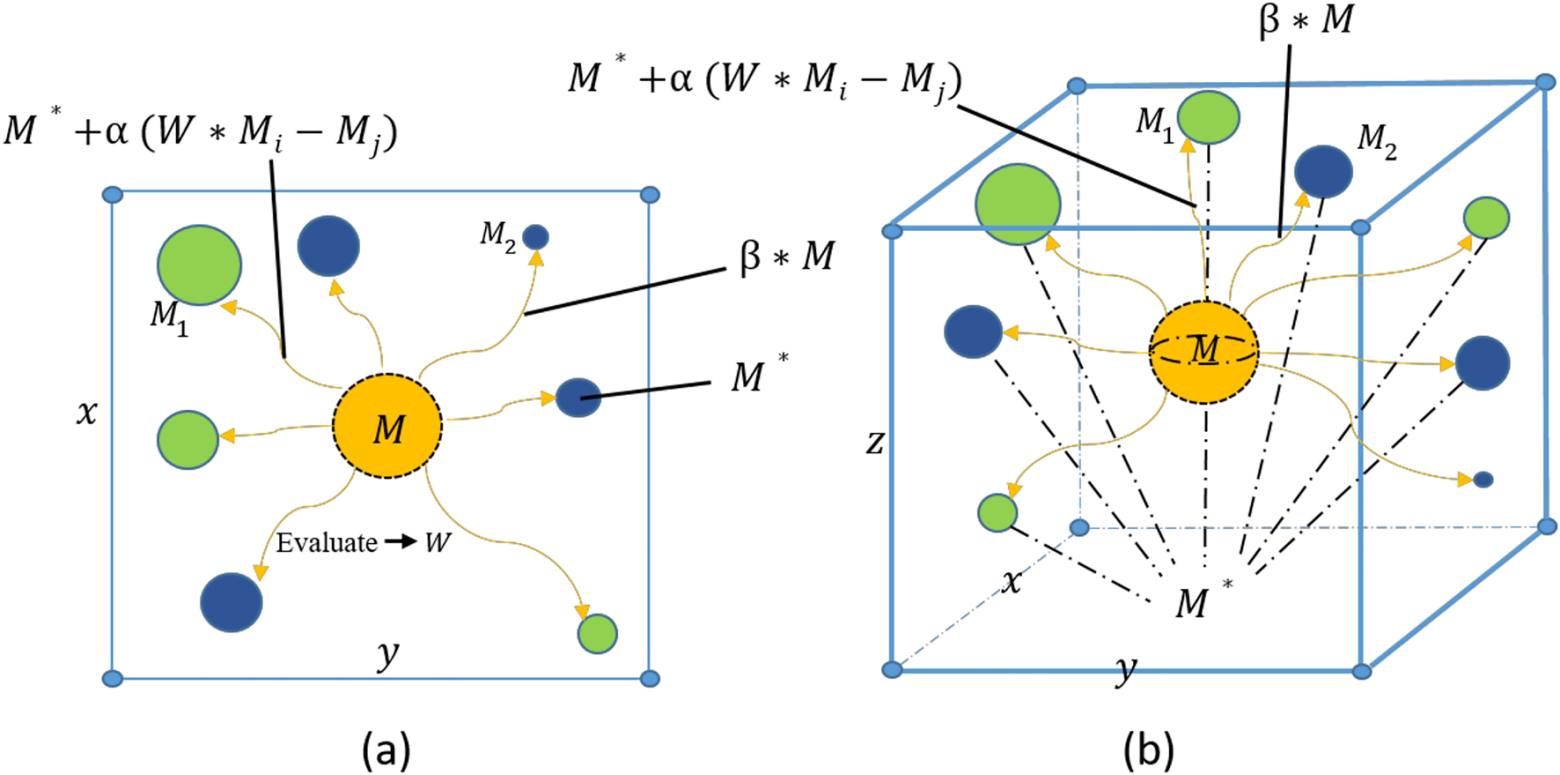
Visualization schematic of SMA in 2D and 3D.

### Seek nutrition

Microbes generally seek nutrition through residual pheromones in the air and along the pathways. Figure 1 is illustrated to understand the visual model of slime molds in seeking nutrition. The asymptotic search behavior of slime molds for nutrition can be formulated by:

1 $$M\left( {t + 1} \right) = \left\{ {\begin{array}{*{20}l} {M^{*} (t) + \alpha \cdot \left( {W \cdot M_{i} (t) - M_{j} (t)} \right),} \hfill & {r < q} \hfill \\ {\beta \cdot M(t),} \hfill & {r \ge q} \hfill \\ \end{array} } \right.$$

where each *M* can be considered as an individual slime mold, and these individuals refresh their positions in compliance with the contemporary optimal individual $M^{*}$, and three related parameters $\alpha$, $\beta$ and *W*. $M_{i}(t)$, $M_{j}(t)$ represent two randomly selected slime individuals, *t* implies the number of iterations, and *W* is a weight learned from the foraging behavior of the slime. $\alpha$ is calculated as a balanced parameter based on the number of iterations, and $\beta$ is a decreasing linear coefficient from 1 to 0. *r* is a random value in [0,1]. *q* is defined by $q=\tanh \vert F_i-F^{*}\vert$. $F_i$ indicates the fitness value of *M*. $F^{*}$ denotes the optimal fitness of the entire iterations.

The distribution of $\alpha$ is in the range $[-a, a]$. The value of *a* is derived from an inverse hyperbolic tangent function regarding the number of iterations, taking values in the range (-1, 1). *a* can be expressed by:

2 $$\begin{aligned} a={\text {arctanh}}\left( -\left( \frac{t}{t_{max}}\right) +1\right) , \end{aligned}$$

where $t_{max}$ is the maximum iteration number. Through this approach, an approximate activity range is assigned to slime molds in each generation.

From Fig. 1, we can see the updated changes in the search position of the slime individuals in the two-dimensional and three-dimensional space. By adjusting the parameters of Eq. (1), the slime molds can investigate in random directions within the search space, forming a free-angle search vector that enhances the probability and capacity of individuals to find the optimal solution. This process stimulates the wrapped venous network formed by the slime bacteria as they approach the food source, searching for everything possible about the food.

### Wrap up nutrition

When the venous network receives enough nutrition information, the bio-oscillator begins to fluctuate information that can regulate the concentration of cytoplasm and biological structure. This process is dedicated to learning about this feedback pattern of slime molds that regulates the structure of biological tissues. *W* in Eq. (1) is expressed mathematically as a positive and negative feedback coefficient between venous tissues and food pheromone concentrations. The definition of *W* is outlined as follows:

3 $$W(i) = \left\{ {\begin{array}{*{20}l} {1 + rand \cdot \log \left( {\frac{{F_{b} - F_{i} }}{{F_{b} - F_{w} }} + 1} \right),} \hfill & {Case - Half} \hfill \\ {1 - rand \cdot \log \left( {\frac{{F_{b} - F_{i} }}{{F_{b} - F_{w} }} + 1} \right),} \hfill & {Otherwise} \hfill \\ \end{array} } \right.$$

where $F_{b}$ means the fitness of the best contemporary individual and $F_{w}$ means the fitness of the worst contemporary individual. The logarithmic function is accustomed to balancing the rate of change of the values and preventing extreme values of the frequency of change. Because of the uncertainty of slime mold’s biological activity, a *rand* factor is attached to model randomness. $Case-Half$ means the case where $F_i$ is at the hand half ranking of the population. Obviously, when the concentration and quality of nutrition are high, the likelihood of a slime mold individual staying in the region for an all-encompassing search becomes greater; when the concentration and quality of nutrition are low, the individual moves to another region.

### Biological oscillator

To better understand the changes in the slime molds of mucilaginous bacteria upon receipt of bio-waves, SMA uses *W*, $\alpha$ and $\beta$ to go for the regulation mechanism of the oscillator. $\alpha$ and $\beta$ are two coefficients that both oscillate randomly within a certain interval and converge to zero. The mutual modulation of these two parameters gives a good indication of the biological stochastic selection behavior of the mucilage. When an individual has found the optimal solution in space, the slime molds may still allocate part of their population to other areas, enhancing the probability of finding the missing food source. It is also an instinct of the organism to find all food sources possible, rather than getting stuck in a localized food area. Furthermore, by adjusting the bidirectional feedback coefficients *W*, the frequency of the bio-wave in the presence of different concentrations of food pheromones can be changed. When good quality food sources founded by venous tissues, *W* will be cranked up to change the cytoplasmic concentration and approach the food source more efficiently; when the quality and concentration of food are not good in some areas, *W* will be lessened to slow down the tissue extension of the region and save energy, so as to choose food sources more efficiently.

The SMA intuitively visualizes the efficient foraging biological activity of slime molds. Nevertheless, the journey to find the best food source is not straightforward and influenced by various factors that may inevitably lead to the trap of local optimal. Therefore, we need to consider adding a number of mechanisms to correct this trend and remedy some of the algorithm’s flaws.

## Multi-chaotic local search operator

The order of the macro universe is built on the disorder of the micro world. This harmony contains underlying laws that existing paradigms cannot describe, explain, or predict. Chaos theory is to study the local uncertainty and the stability of the whole, the order hidden in the unpredictable phenomenon. Most scenarios we encounter in reality are non-linear systems that cannot be solved by conventional experience and theory, with complex interactions between elements within the system that are difficult to quantify. Chaotic systems generally have the following three typical characteristics:

1. If a system makes a chaotic motion, the orbit of the system is disproportionately sensitive to tiny changes in the initial state, or a small change produced in one part of the system can lead to a violent reaction in the whole system.
2. Chaotic systems have fractal properties, that is, the system is irregular in its overall structure from the beginning, but the degree of irregularity of the system is repetitive at different scales.
3. Systems always exhibit a state of mutual antagonism and coupling between static equilibrium features and the tendency to fall into non-predetermined patterns.

### Chaotic maps

**Figure 2:**
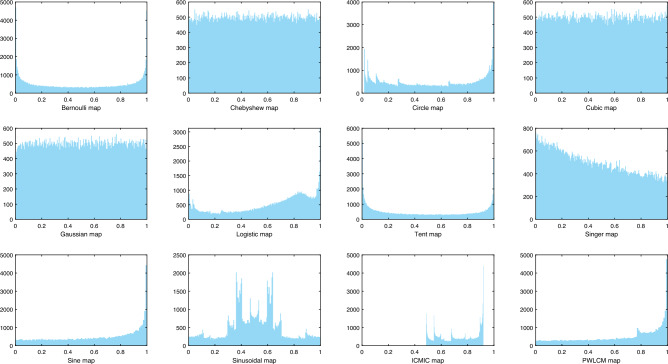
The histogram of the distribution produced by 12 different chaotic maps.

Chaotic map can be conceived as a function used to generate random chaotic arrays. In the field of evolutionary computation, algorithms often require pseudo-random number generators for population initialization, but sometimes the results are not satisfactory. It is found that due to the unpredictability and ergodicity of chaotic maps, better results are attained by replacing pseudo-random generators with chaotic maps^39^. In our study, 12 representative chaotic maps are chosen, taking one of the well-known maps Chebyshev as an example, whose chaotic map formula is:

4 $$\begin{aligned} x_{n+1}=\cos \left( O \arccos x_{n}\right) , x_{n} \in [-1,1], \end{aligned}$$

where *x* and *n* belongs to the set of integers. *O* denotes the order of the Chebyshev map. When *O* is greater than or equal to 2, no matter how approximate the initial value is selected, the resulting iterated sequence has no correlation, i.e., the system is in chaos. Figure 2 shows the histogram of the distribution produced by 12 different chaotic maps.

### Maximum Lyapunov exponent

The maximum Lyapunov exponent is an essential quantitative indicator for measuring and determining whether a non-linear system is undergoing chaotic motion. For chaotic systems, trajectories initiated by two enormously close initial variables produce exponential separation over time, and the MLE is defined to quantify the quantity that describes this separation rate.

Assume a 1-dimensional discrete dynamical system: $x_{n+1}=\textrm{f}\left( x_{n}\right)$. After *n* iterations, whether the initial two points are separated or close in space depends on the derivative $\left| \frac{d f (x_{n})}{d x_{n}}\right|$. For an initial variable point $x_{0}$, we set the change in position caused by each iteration to have an exponential separation rate of $\Lambda$. Then the initial distance $\Delta$ between the two points after iteration becomes:

5 $$\begin{aligned} \Delta e^{n\Lambda }=\left| f^{n}\left( x_{0}+\Delta \right) -f^{n}\left( x_{0}\right) \right| . \end{aligned}$$

Taking the limits $\Delta \rightarrow 0$, $n \rightarrow \infty$, then Eq. (5) can be deformed to

6 $$\begin{aligned} \Lambda =\lim _{n \rightarrow \infty } \lim _{\Delta \rightarrow 0} \frac{1}{n} \ln \left| \frac{f^{n}\left( x_{0}+\Delta \right) -f^{n}\left( x_{0 }\right) }{\Delta }\right| . \end{aligned}$$

The above equation can be simplified as:

7 $$\begin{aligned} \Lambda =\lim _{n \rightarrow \infty } \frac{1}{n} \sum _{i=0}^{n-1} \ln \left| \frac{d f(x)}{d x}\right| _{x=x_{0}}. \end{aligned}$$

From this we can generalize this definition to all problems and obtain the defining equation for the maximum Lyapunov exponent as:

8 $$\begin{aligned} \Lambda =\lim _{t \rightarrow \infty } \lim _{\delta Y_{0} \rightarrow 0} \frac{1}{t} \ln \frac{\vert \delta \textbf{Y} (t)\vert }{\vert \delta \textbf{Y} _ {0}\vert }. \end{aligned}$$

where $\delta \textbf{Y} (t)$ and $\delta \textbf{Y} _ {0}$ represent the trajectories of motion caused by two initial values in the dynamical system, respectively. It is intuitive to see that if $\Lambda$ is greater than or equal to 0, that means that no matter how close the initial two tracks are, the difference in their trajectories will be exponentially magnified in space with time. So we can draw two conclusions: (1) If a system has at least one MLE greater than 0, the system does the chaotic motion. (2) The MLE of periodic motion or steady state must be at least non-positive.

According to the above definitions, we can obtain the MLE of each chaotic map separately, and the value of numbers are summarized in Table 1. We consider the MLE as a fundamental characteristic of the different chaotic sequences and will discuss later how this fundamental property can be incorporated into the consideration of the local operators.

**Table 1:** The maximum Lyapunov exponent values of chaotic maps.

Name	Bernoulli map	Chebyshev map	Circle map	Cubic map	Gaussian map	Logistic map
MLE	0.655	0.948	0.295	0.667	2.659	0.692
Name	Tent map	Singer map	Sine map	Sinusoidal map	ICMIC	PWLCW
MLE	0.683	0.423	0.687	0.567	4.719	0.190

### Chaotic local search operator

Meta-heuristic algorithms are an interactive association of global explore strategy and local search operator in essence. The research and improvement of search strategies over the years are essential to allow the algorithm to explore the solution space more rationally and get free from the problem of being induced by local optima. Following the chaotic properties introduced earlier, the offspring generated by a chaotic map are randomly and irregularly varying. If an algorithm is assigned enough computational time and computational resources, we can approximately believe that the algorithm is able to travel the whole search area and find the target solution we want. But once the problem is of high dimensionality and high computational complexity, it will require a huge amount of resources and optimization time, which is not in line with the aim of computer science to pursue efficiency. Thus, the utilization of chaotic search operators in small search spaces and specific phases can be a significant way to enhance search performance. To date, this operator has been applied to extensive algorithms for global search strategies, and many fruitful achievements have been realized.

Alatas et al.^40^ proposed an improved harmony search algorithm by replacing the random sequence in the initialization of harmony search algorithm with each of seven different chaotic maps and tests the performance of seven chaotic combination algorithms in solving optimization problems. It was found that this approach improved the performance of the algorithms in global search. Yuan et al.^41^ combined the quantum thinking and chaotic local search in traditional artificial bee colony algorithm. In contemporary iterations, the swarm performs a disorderly search around the vicinity of the current best food source found, which can well circumvent the algorithm being captured by the local optimum through the jumpiness of chaos. Gao et al.^42^ reformed the local search-based differential evolution by chaos, embedding multi-chaos local search operator based on success probability in the mutation process. It effectively improves the inherent defects of most differential evolution variants, namely premature convergence and unstable performance. In addition, four chaotic variants are proposed based on different applications of chaotic local search, and the effectiveness of multi-chaos is demonstrated on a sufficient number of test problems.

Many examples prove that chaotic local search has a comprehensive and successful application in meta-heuristic algorithms. Scholars have used chaotic local search in a variety of ways to help algorithms improve the capacity of exploring and exploiting the search space, avoid the interference of local optima, and perform efficient and accurate convergence behavior.

## MLE-based multiple chaotic SMA

In this section, we specify the sources of inspiration for MCSMA and the operation mechanism of the algorithm. The three questions of how to include a chaotic local search in SMA, which way to call chaotic maps, and how to improve the local operator are explained in detail. The flowchart and pseudo-code of MCSMA are also introduced.

### Inspiration

In the previous section, we have described the biological mechanisms and mathematical modeling process of SMA in detail. The biological activities of single-celled organisms appear to be disorderly and random, but there are also characteristic laws behind them. For a microscopic individual such as a slime mold, the most challenging task is how to find food information accurately in a vast space. Similarly, for a good algorithm, the most critical problem to be solved is how to efficiently find that optimal solution over the entire solution space^8,43^. In SMA, the individuals of the population update and judge their position by employing positive and negative feedback coefficients. From Eq. (1), we can assume that the individuals perform two kinds of ordered activities in the solution space under the adaptation of the feedback parameters. When no food information is detected temporarily, individuals adjust their corresponding positions to each other, move and search towards the region of the angle between two individuals, or possibly continue exploring along the direction of their own vector. However, in this process, the globally optimal solution we need may be hidden in the vacant solution space of these two alternative paths. We cannot rule out this possibility, so the question of how to allow individuals of the population to search more fully through the entire solution space is an urgent problem to be solved. The tremendous advantage of chaotic motion is that completely disordered motion in a given region can significantly compensate for the algorithm’s weakness in local exploration. From the perspective of exploration and exploitation of the overall search space, the slime mold individuals in SMA demonstrate satisfactory exploration capabilities, allowing the population to explore all potential regions. However, SMA lacks strong exploitation of specific regions, which increases the risk of search stagnation and premature convergence. Considering the advantages of chaotic search, we contemplate how to incorporate chaotic local search operator into the feedback mechanism of slime individuals for food information. By incorporating chaotic local search operators to perturb individual trajectories in an unordered manner, we aim to enhance the algorithm’s specialized exploitation capabilities in promising regions, aiding in achieving desired optimization results.

### MCSMA

Herein, we present an ameliorated algorithm MCSMA based on the predatory behavior of slime molds and multiple chaotic local operators for the first time. The underlying algorithm SMA has proven to be a strong global search algorithm. As the number of iterations increases, the distribution of populations in the search space exhibits a cross-searching motion track. Especially in the earlier phase of the iterations, the renewal of individual positions fluctuates very sharply in the early stages because of the parameters $\alpha$ and *W*. Thus SMA can rapidly converge at an early stage and explore a significant portion of the entire exploration space. Subsequent iterations of individuals converge in regions that are likely to be globally optimal and conduct disordered exploration. This ensures the global search capability of the algorithm. But when $r \ge q$, the population will engage in selective behavior, with some of the slime heading towards other regions, and some other individuals keeping their original direction for oscillatory search. Along this search trajectory, there is a possibility that the optimal solution may be neglected in space, or captured by a local optimum in a small region. We therefore consider the insertion of a powerful exploitation mechanism in this process, namely the chaotic local operator.

In a local operator using a single map, the operator generates the next generation of new individuals, employing a contemporary globally optimal individual $E_{k}$. The formula for chaotic search can be expressed as:

9 $$\begin{aligned} \widetilde{E_{k}}=E_{k}+\phi (V_{U}-V_{L})(\omega _{k}-0.5) \end{aligned}$$

where $\widetilde{E_{k}}$ denotes the potential individual to replace the contemporary globally optimal. $V_{U}$ is the upper bound vector of the population, and $V_{L}$ is the lower bound vector. $\phi$ can be understood as a spatial scale that can represent the chaotic search. $\omega _{k}$ represents the distribution variables generated by the chaotic map in this iteration. In the contemporary iteration, if $\widetilde{E_{k}}$ has a superior fitness than $E_{k}$, it renews $E_{k}$ in the next generation of search behavior. This renewal reflects the behavior of an individual, which performs a chaotic expansion in space.

From^42^, we learn that using multiple chaotic maps can often achieve better results than using single chaos. The combinations of plural chaotic maps can incorporate different dynamical properties and keep the dynamics changing in space. There are various combinations in the algorithm including parallel, sequential, and permutation, and so on. In this study, we use the traditional roulette wheel idea, where the selection probability of each individual is proportional to its fitness value, and choose 12 chaotic maps with different dynamics to form a probabilistic roulette wheel. However, different from the preceding methods, we take the ideology of meritocracy as the guide to determine the chaotic map used in the iterations. For a given problem, if a particular chaotic map selected by the meritocracy improves the algorithm more in a certain iteration, then we can assume that this chaotic map may have good compatibility with the problem and its dynamics can better help the algorithm access this problem. In the next iteration, the probability of selecting this superior map in the previous generation is incremented to find the most suitable chaotic search operator.

Using this roulette strategy based on the principle of meritocracy enables the algorithm to find the best chaotic operator to solve the test problem quickly. However, parts of the chaotic maps may be given great weight in the initial iteration, resulting in a lack of competition for other chaotic maps in subsequent iterations. This will lead to the algorithm missing some relatively superior maps and suffering from the waste of computational resources and poor robustness. Based on this consideration, we focus on the maximum Lyapunov exponent, a property of chaos itself, aiming to investigate the best chaotic local operator from the fundamental properties of chaotic maps. A probabilistic compensation mechanism is introduced to give the operator a second chance to choose the suitable map based on a meritocratic roulette wheel selection.

The maximum Lyapunov exponent is an index that measures the tendency of a chaotic system to move over time. Twelve different chaotic maps have their MLE values. We can define a correlation coefficient $C_{i j}$ based on the MLE, formulated as:

10 $$\begin{aligned} C_{i j}=\frac{1}{\tan \left( \frac{\pi }{4}+\frac{\pi }{4} \times \mid L_{i}- L_{j} \mid \ / \ L_{ max }\right) \times rand} \end{aligned}$$

where *rand* is a random coefficient; $L_{i}$ and $L_{j}$ represent the MLE values of two distinct random chaotic mappings. $C_{i j}$ is the correlation coefficient normalized to the MLE values of any two chaotic maps with values distributed in (0, 1). Based on this, we can construct a 12*12 matrix as shown as follows:

11 $$\begin{aligned} C = \begin{bmatrix} 1 &{} C_{1,2} &{} \cdots &{} C_{1,12} \\ C_{2,1} &{} 1 &{} \cdots &{} C_{2,12} \\ \vdots &{} \vdots &{} \ddots &{} \vdots \\ C_{12,1} &{} C_{12,2} &{} \cdots &{} 1 \\ \end{bmatrix}_{12*12.} \end{aligned}$$

**Figure 3:**
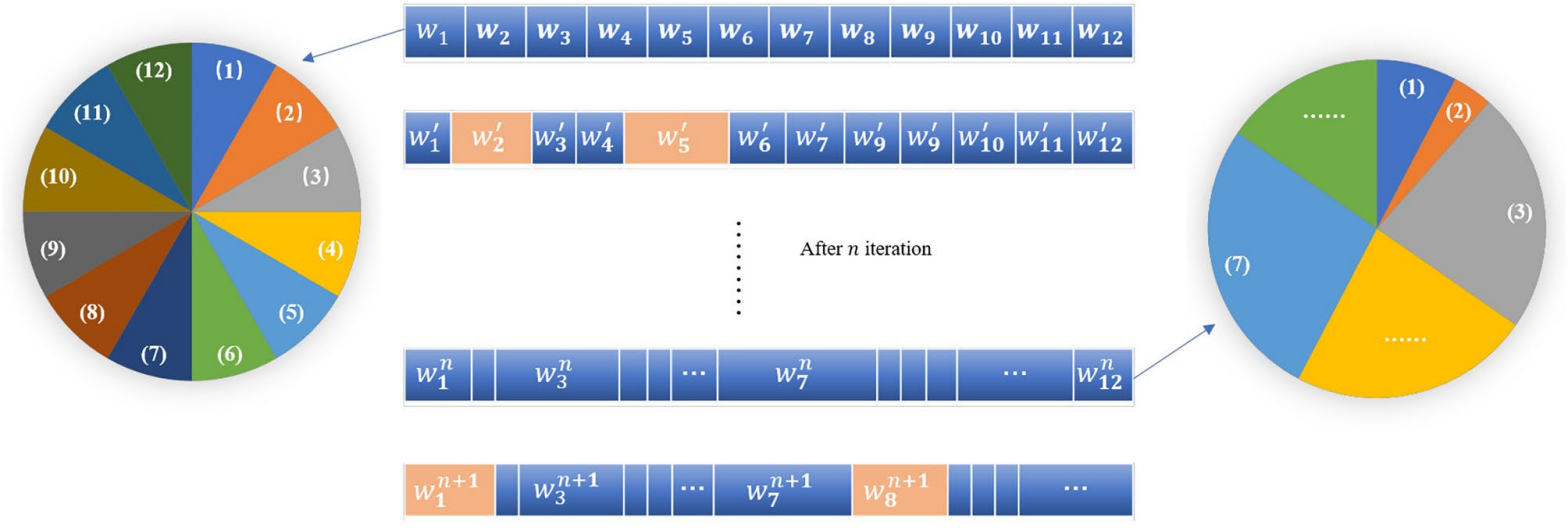
The process of adjusting the span of the weighted wheel.

This matrix represents the nature of the association between the individual chaotic maps. Once we have chosen the most appropriate contemporary chaotic map, it is reasonable to believe that the chaotic map with the closest value of its MLE also has a better improvement on the target problem. Therefore, we give the optimal and sub-optimal maps a conditioning weight in the probability adjustment of roulette selection. The adjustment weights $W_{k}$ are defined as follows:

12 $$\begin{aligned} W_{k}=\xi \cdot C_{k, index(s)}^{s} \end{aligned}$$

where *s* is a parity ordinal number that takes the value 1 or 2. *index*(*s*) represents the index position in the roulette wheel of the chosen optimal and sub-optimal maps. $\xi$ is a distribution of chaotic weights in interval (0, 2). When adjusting the weights for the optimal chaotic map, as it is the highest priority map, *s* takes the value of 1. When adjusting the weights for the sub-optimal map, as viewed as a sub-optimal chaotic choice, this map is compensated with a smaller adjustment weight, *s* takes the value of 2. Figure 3 illustrates this weight adjustment process. Relying on adjusting *s* and the correlation $C_{i j}$ values, we can rationalize the span of roulette weights to select the most appropriate chaotic map and avoid premature formulation of the map map by the chaotic local operator.

**Figure 4:**
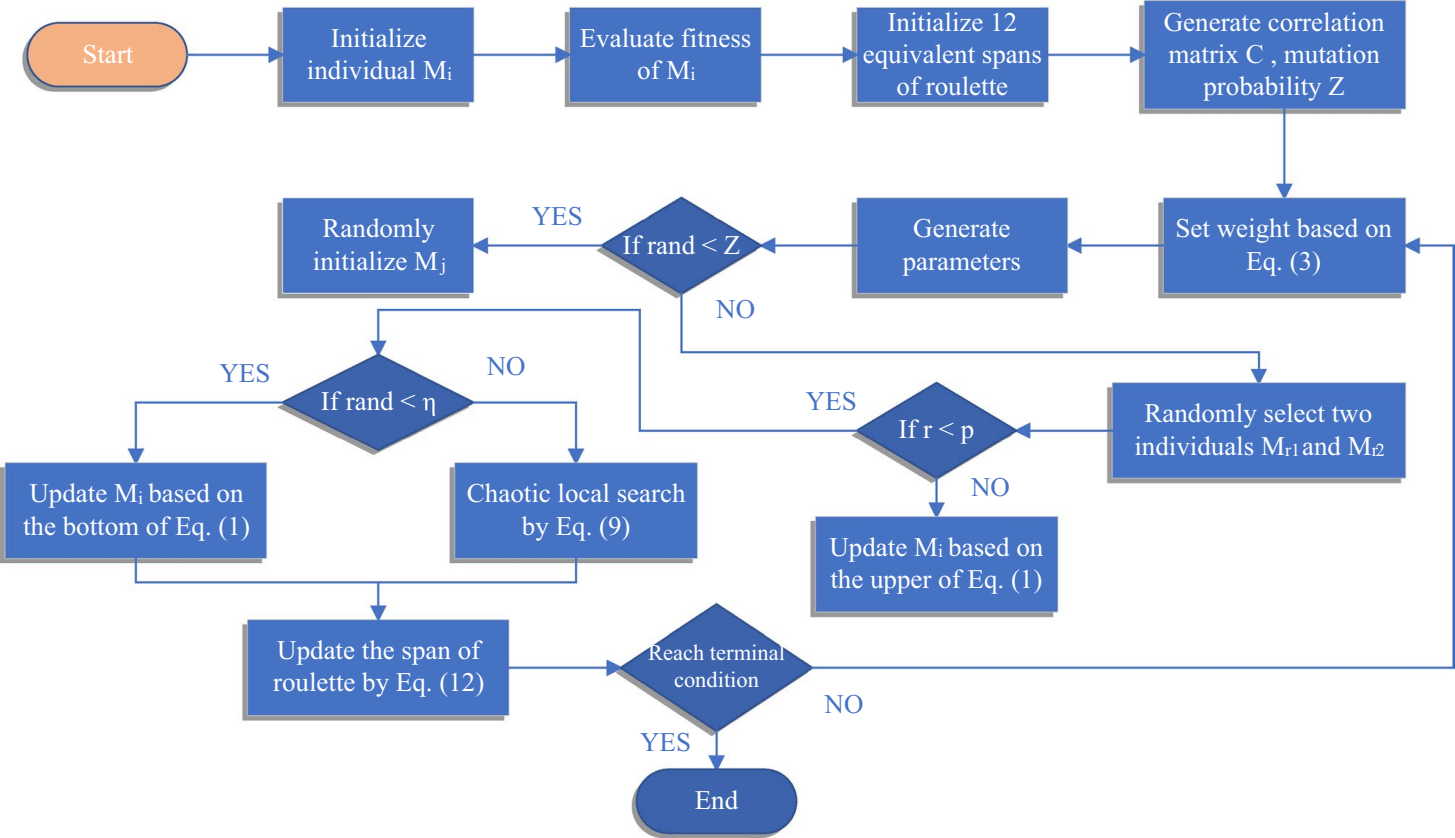
A general flow chart of MCSMA.

The schematic pseudo-code of MCSMA is illustrated in Algorithm 1. The general flowchart of MCSMA is depicted in Fig. 4. The entire operation flow of MCSMA can be generally summarized as follows:

1. MCSMA starts to generate the slime molds $M_i$ and evaluate the fitness.
2. Initialize twelve equivalent spans of roulette and generate correlation coefficient matrix *C*, mutation probability *z*.
3. According to the feedback mechanism of slime molds, the algorithm selects and updates $M_i$ based on Eq. (1).
4. When the algorithm matches the pre-defined scenario, MCSMA enters the phase of chaotic local search and utilizes roulette wheel selection to select the chaos operator by Eq. (9).
5. Update the span of roulette by Eq. (12) and re-tune the chaotic local operator.
6. Repeat the above steps until reaching the terminal condition.

In summary, we establish a selection weight adjustment mechanism based on the connection of MLE to optimize the chaotic local operator. Based on the kinematic properties of chaotic behavior, a plausible screening mechanism is established to guide the search pattern of the local operator. In addition, considering the search behaviors and trajectories of slime mold individuals in MCSMA, we expect to explore a general way to refine the underlying search logic of the algorithm and guide the algorithm to improve its reliability throughout the search process^44,45^.

### Ethical approval

No human or animal subjects were involved in this experiment.
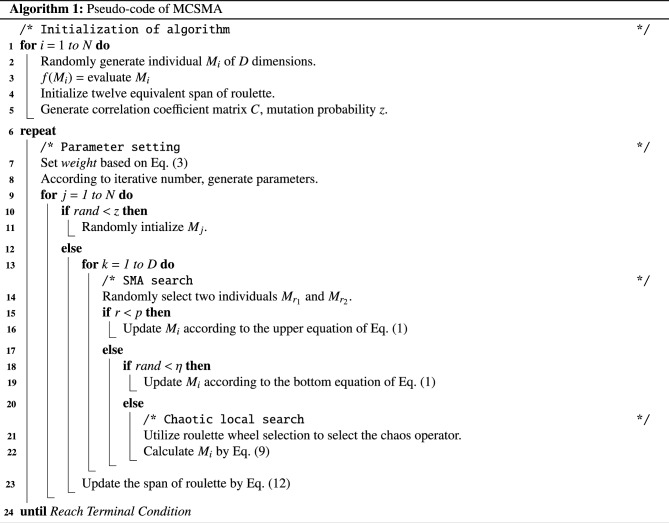

## Experimental analysis

To evince the capability of the proposed algorithm MCSMA, numerous test sets were selected for experimentation. To validate the effectiveness of MCSMA on different kinds of optimization problems in different dimensions, 29 problems from CEC2017 were elected to run experiments and data analysis. CEC2017 contains 2 unimodal problems (F1, F2), 7 multimodal problems (F3-F9), 10 mixed-state problems (F10-F19) and 10 combinatorial optimisation problems (F20- F29). The Wilcoxon rank-sum test, the convergence curve graph, and the box-and-whisker chart were applied to analyze the experimental data in a multifaceted way. Meanwhile, for the propose of examining the algorithm’s ability to handle real-world problems, we experiment with 22 real-world problems from CEC2011 and ANN training.

### Experimental set up

For CEC2017: The proportion of population *N* is regulated by 100, The dimension of problems *D* is designed with three sets of data, i.e., 30, 50, and 100, respectively. This operation is to examine the effectiveness of MCSMA when facing high-dimensional problems and whether it have defects such as overfitting. The maximum number of fitness evaluation is determined as $10000*D$. To obtain a more credible experimental result, we position the number of independent runs at 51. The search range is arranged in the interval $[-100, 100]$.

For CEC2011: The size of population *N* is regulated by 100. Because the optimization model is learned from the actual problem, each problem has its adapted dimension. The specific situation is summarized in Table 7. We set 30 as the number of runs because the optimization time required for the test problems is time-consuming.

The experimental equipment is configured with 16GB RAM and a 3.00 GHz Intel(R) Core(TM) i5-7400 CPU, and the test platform was MATLAB.

### Comparison analysis on CEC2017

In this set of benchmark experiments, we select HHO^46^, WOA, MFO^47^, SSA^48^, SCA^49^ and GLPSO^50^ as the comparison targets in addition to the underlying algorithm SMA. These meta-heuristic algorithms are inspired by the biological phenomena in nature or mathematical laws in recent years. For example, HHO simulates the teamwork and chase patterns of a falcon hunting a rabbit, and has excellent advantages in solving single-target problems. We expect to test the performance of MCSMA under different circumstances with these algorithms that possess different comparative advantages. The particular parameter setting is listed in Table 2.

**Table 2:** The parameter settings of the comparative algorithms.

Algorithms	Settings
MCSMA	$\eta =0.5;z=0.03$
SMA	$z=0.03$
HHO	$E0\in (-1,1);E1=2$
WOA	$I=1;a_{1}\in [2,0];a_{2}\in [-2,-1]$
MFO	$I=1;a\in [-1,-2]$
SSA	$c_{2},c_{3}$ randomly located in [0,1]
SCA	$A=2$
GLPSO	$c_{1}=1.49618;O=0.7298;pm=0.1$

The first assessment criterion is the Wilcoxon rank-sum test, which is a two-sample *t*-test^51^. The median confidence interval is set to $95\%$ to infer the distribution of the overall values when comparing two mutually independent data sets. Table 3 exhibits the final compared experimental data of the seven control groups in 30 dimensions. “MEAN” describes the median of the data sample set. “STD” refers to the standard deviation, which is an important statistic to measure the degree of dispersion of the data. The Wilcoxon test generally has three types of ranking comparison results: “$+$”, “$\approx$”, and “−”, indicating MCSMA performs better, tied, or worse than it comparison algorithms, respectively. In this table, the symbols “*W*/*T*/*L*” indicate the total number of three results of win, tied, and lose, respectively. The data group in bold means that the data is the optimal value for the same group under this test function. The comparison result between MCSMA and the basic algorithm SMA is 19/6/4, which indicates that this improved method we proposed has a great advancement on the algorithm. The comparison results between MCSMA and the other six meta-heuristic algorithms are 28/1/0, 29/0/0, 29/0/0, 20/3/6, 29/0/0, and 16/7/6, respectively. This positive result indicates that MCSMA achieves superior performance on most of the tested problems. We compare the *p*-values obtained from the Wilcoxon rank-sum test with the significant level of 0.05 to determine the presence of significant differences in the experimental results. Table 4 provides a detailed comparison of *p*-values for the 30-dimensional case. The symbols following the specific *p*-values represent the final outcome of the experimental comparison. It is evident that the obtained *p*-values compared with the original and other powerful meta-heuristic are substantially lower, indicating a significant improvement in the performance. In addition, we examine the stability and performance of MCSMA in both 50 dimensions and 100 dimensions, summarized in Table 5 and Table 6, respectively. It is noticeable that the MCSMA’s prime value and the number of wins increases as the dimensionality magnifies. This trend proves the stability of MCSMA on high-dimensional problems. When other algorithms fall into overfitting or local optimum, MCSMA still maintains stronger robustness.

**Figure 5:**
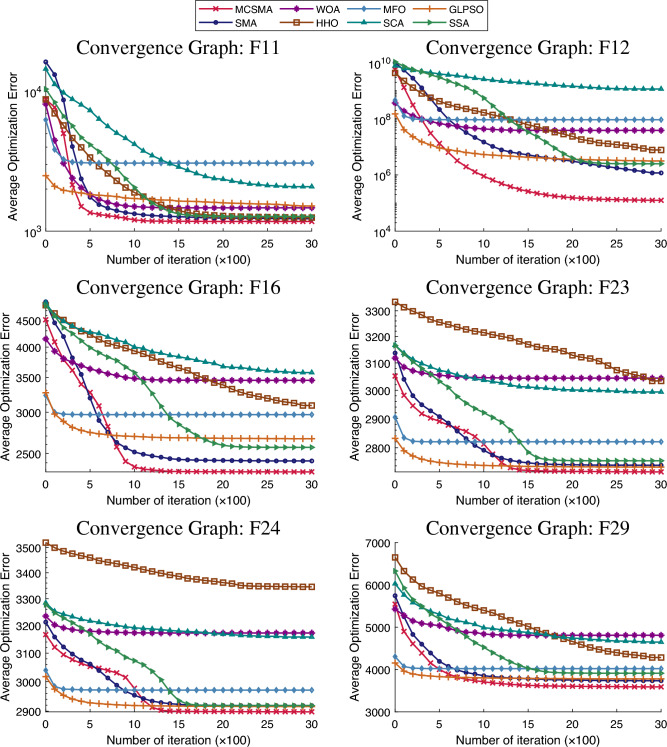
The exhibition of convergence comparison on Functions 11, 12, 16, 23, 24, and 29.

The second assessment criterion is the convergence diagram of different algorithms^52^. This test is mainly to visually match the convergence speed with performance of the algorithm. As displayed in Fig. 5, the horizontal coordination represents the number of evaluations on different function problems, and the vertical coordination represents the average optimization error that the algorithm can achieve. We choose six different types of functions F11, F12, F16, F23, F24, and F29 to show the convergence ability of MCSMA. The curve slope of MCSMA is the smallest in the first period of evaluation, which indicates that MCSMA is able to converge to desired solutions at a faster rate. The lowest point of the MCSMA curve is always the smallest of the six functions, verifying that MCSMA has the capability of grasping the best solution. In summary, MCSMA possesses a fast convergence speed and excellent performance.

**Figure 6:**
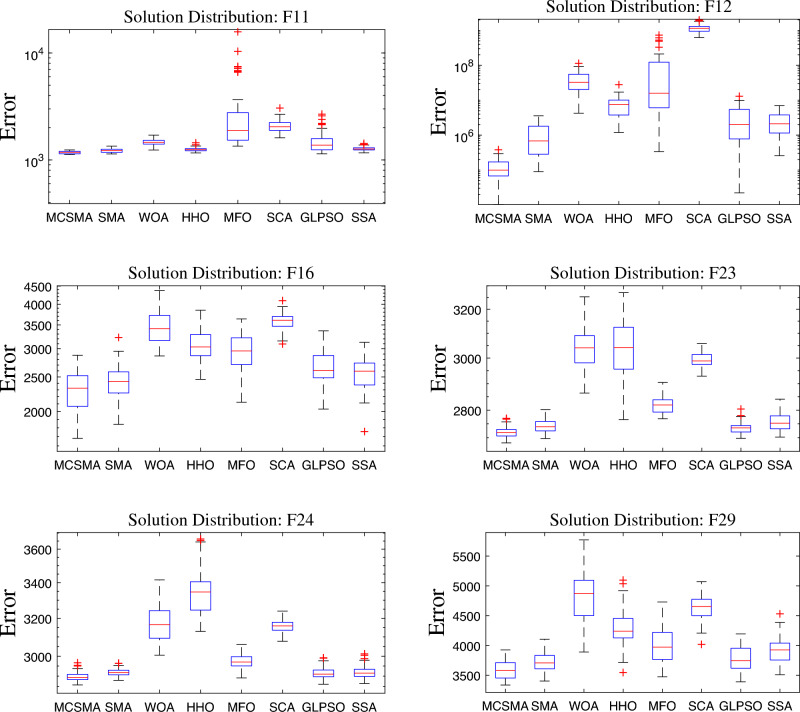
The exhibition of box-and-whisker diagrams on Functions 11, 12, 16, 23, 24, and 29.

The third evaluation criterion is the box-and-whisker chart. This chart is mainly used to evaluate the quality of the solutions obtained by the algorithm. It can visually furnish the distribution characteristics between different data groups and manifest their differences. As displayed in Fig. 6, the lines inside the box depict the median of the data, and the upper and lower edges represent the quartile spacing boxes. The smaller spacing between the edges of the box indicates that the overall distribution of the data is in a confidence interval, which suggests the better robustness of the algorithm. The upper and lower black lines outside the box refer to the maximum and minimum values of the algorithm’s solution, respectively. The box is located in the lower space, suggesting the better solution quality of the algorithm. The red crosses are the outliers in the data, and the fewer outliers indicate the more stable performance of the algorithm and the higher confidence of the data. Since outliers often have an opposing influence on the distribution and characteristics of a set of data and affect the analysis and judgment of the data, we have to consider this factor cautiously. On the six test functions we selected, it is clear that MCSMA consistently has the lowest spatial position and the smallest spacing. This illustrates that MCSMA has the highest solution quality and the strongest stability in this control group.

In summary, we carry out a series of comparison experiments under different dimensions on the CEC2017 standard test set and analyze the experimental results using three distinct data evaluation methods^53^. The analysis shows that MCSMA is quite competitive in terms of the overall solution quality, convergence rate, performance stability and robustness of the algorithm.

**Table 3:** The CEC2017 experimental results of MCSMA and other comparative algorithms in 30 dimensions.

	MCSMA	SMA	HHO	WOA
	MEAN STD	MEAN STD	MEAN STD	MEAN STD
F1	9.401E+03 ± 7.717E+03	7.500E+03 ± 6.498E+03 $\approx$	8.066E+06 ± 1.765E+06 +	2.355E+06 ± 1.934E+06 +
F2	3.015E+02 ± 5.027E−01	3.001E+02 ± 7.370E−02 −	9.796E+02 ± 3.608E+02 +	1.593E+05 ± 7.032E+04 +
F3	4.948E+02 ± 1.382E+01	**4.907E+02** ± **1.081E+01** −	5.117E+02 ± 2.457E+01 +	5.367E+02 ± 3.795E+01 +
F4	5.714E+02 ± 2.010E+01	5.826E+02 ± 2.014E+01 +	7.168E+02 ± 2.972E+01 +	7.665E+02 ± 5.179E+01 +
F5	6.022E+02 ± 7.549E−01	6.008E+02 ± 6.172E−01 −	6.563E+02 ± 5.915E+00 +	6.673E+02 ± 9.256E+00 +
F6	**8.034E+02** ± **1.943E+01**	8.143E+02 ± 2.523E+01 +	1.206E+03 ± 7.732E+01 +	1.234E+03 ± 8.860E+01 +
F7	8.765E+02 ± 2.086E+01	8.871E+02 ± 2.264E+01 +	9.511E+02 ± 1.930E+01 +	1.029E+03 ± 4.884E+01 +
F8	**1.335E+03** ± **8.465E+02**	2.294E+03 ± 1.174E+03 +	5.911E+03 ± 6.254E+02 +	8.003E+03 ± 3.541E+03 +
F9	3.824E+03 ± 5.848E+02	4.041E+03 ± 6.560E+02 +	5.297E+03 ± 6.219E+02 +	5.946E+03 ± 7.043E+02 +
F10	**1.172E+03** ± **3.527E+01**	1.226E+03 ± 5.640E+01 +	1.250E+03 ± 5.166E+01 +	1.469E+03 ± 1.157E+02 +
F11	**1.249E+05** ± **7.938E+04**	1.181E+06 ± 1.167E+06 +	7.758E+06 ± 4.810E+06 +	3.871E+07 ± 2.494E+07 +
F12	2.549E+04 ± 2.525E+04	2.663E+04 ± 2.484E+04 $\approx$	2.149E+05 ± 1.391E+05 +	1.468E+05 ± 1.036E+05 +
F13	1.140E+04 ± 7.108E+03	3.835E+04 ± 1.972E+04 +	3.008E+04 ± 2.755E+04 +	7.566E+05 ± 9.071E+05 +
F14	1.866E+04 ± 1.559E+04	2.723E+04 ± 1.304E+04 +	5.384E+04 ± 4.722E+04 +	6.047E+04 ± 3.826E+04 +
F15	**2.305E+03** ± **3.019E+02**	2.419E+03 ± 2.936E+02 +	3.091E+03 ± 3.279E+02 +	3.454E+03 ± 3.710E+02 +
F16	2.045E+03 ± 1.811E+02	2.146E+03 ± 1.853E+02 +	2.574E+03 ± 2.800E+02 +	2.474E+03 ± 2.474E+02 +
F17	2.319E+05 ± 1.984E+05	3.367E+05 ± 2.837E+05 +	5.534E+05 ± 5.243E+05 +	2.617E+06 ± 2.625E+06 +
F18	2.003E+04 ± 1.993E+04	3.514E+04 ± 1.963E+04 +	1.369E+05 ± 9.906E+04 +	3.139E+06 ± 2.089E+06 +
F19	2.432E+03 ± 1.915E+02	**2.419E+03** ± **1.563E+02** $\approx$	2.692E+03 ± 1.844E+02 +	2.703E+03 ± 1.726E+02 +
F20	2.371E+03 ± 1.949E+01	2.393E+03 ± 2.514E+01 +	2.526E+03 ± 4.017E+01 +	2.556E+03 ± 6.976E+01 +
F21	5.088E+03 ± 1.104E+03	5.228E+03 ± 9.297E+02 $\approx$	5.679E+03 ± 2.249E+03 +	6.810E+03 ± 2.066E+03 +
F22	**2.720E+03** ± **1.755E+01**	2.742E+03 ± 2.236E+01 +	3.034E+03 ± 1.016E+02 +	3.044E+03 ± 9.282E+01 +
F23	**2.898E+03** ± **2.207E+01**	2.919E+03 ± 2.030E+01 +	3.345E+03 ± 1.323E+02 +	3.172E+03 ± 1.010E+02 +
F24	2.888E+03 ± 1.804E+00	**2.888E+03** ± **7.825E+00** −	2.906E+03 ± 1.906E+01 +	2.941E+03 ± 2.984E+01 +
F25	**4.350E+03** ± **3.559E+02**	4.585E+03 ± 2.521E+02 +	6.747E+03 ± 1.294E+03 +	7.666E+03 ± 1.121E+03 +
F26	**3.212E+03** ± **1.068E+01**	3.212E+03 ± 1.163E+01 $\approx$	3.315E+03 ± 5.812E+01 +	3.363E+03 ± 9.531E+01 +
F27	3.237E+03 ± 2.829E+01	3.238E+03 ± 3.994E+01 $\approx$	3.238E+03 ± 2.491E+01 $\approx$	3.305E+03 ± 4.597E+01 +
F28	**3.590E+03** ± **1.439E+02**	3.735E+03 ± 1.794E+02 +	4.284E+03 ± 3.191E+02 +	4.810E+03 ± 4.492E+02 +
F29	**1.148E+04** ± **4.274E+03**	1.621E+04 ± 4.846E+03 +	9.621E+05 ± 5.029E+05 +	8.941E+06 ± 6.614E+06 +

*W*/*T*/*L*	${-}{-}/{-}{-}/{-}{-}$	19/6/4	28/1/0	29/0/0
	MFO	SSA	SCA	GLPSO
	MEAN STD	MEAN STD	MEAN STD	MEAN STD
F1	7.870E+09 ± 5.290E+09 +	**5.010E+03** ± **5.900E+03** −	1.203E+10 ± 1.565E+09 +	3.641E+04 ± 1.220E+05 −
F2	8.288E+04 ± 5.657E+04 +	**3.000E+02** ± **1.180E-08** −	3.569E+04 ± 7.964E+03 +	2.080E+04 ± 1.992E+04 +
F3	8.184E+02 ± 3.305E+02 +	4.950E+02 ± 1.840E+01 $\approx$	1.443E+03 ± 2.640E+02 +	5.055E+02 ± 2.984E+01 +
F4	6.896E+02 ± 4.294E+01 +	6.380E+02 ± 4.780E+01 +	7.756E+02 ± 2.159E+01 +	**5.674E+02** ± **2.055E+01** $\approx$
F5	6.248E+02 ± 1.183E+01 +	6.370E+02 ± 1.260E+01 +	6.487E+02 ± 4.466E+00 +	**6.002E+02** ± **1.124E-01** −
F6	1.014E+03 ± 1.292E+02 +	8.660E+02 ± 4.060E+01 +	1.122E+03 ± 4.005E+01 +	8.303E+02 ± 2.286E+01 +
F7	9.745E+02 ± 3.910E+01 +	9.290E+02 ± 3.840E+01 +	1.046E+03 ± 1.859E+01 +	**8.668E+02** ± **1.686E+01** −
F8	5.989E+03 ± 1.876E+03 +	3.560E+03 ± 1.460E+03 +	5.422E+03 ± 1.202E+03 +	1.373E+03 ± 3.694E+02 +
F9	5.180E+03 ± 6.915E+02 +	5.020E+03 ± 6.570E+02 +	8.185E+03 ± 3.407E+02 +	**3.664E+03** ± **4.481E+02** $\approx$
F10	3.073E+03 ± 2.791E+03 +	1.270E+03 ± 5.540E+01 +	2.082E+03 ± 2.717E+02 +	1.514E+03 ± 3.850E+02 +
F11	9.258E+07 ± 1.646E+08 +	2.500E+06 ± 1.680E+06 +	1.154E+09 ± 2.865E+08 +	3.147E+06 ± 2.980E+06 +
F12	5.371E+05 ± 1.318E+06 +	1.150E+05 ± 8.320E+04 +	3.783E+08 ± 1.618E+08 +	**2.137E+04** ± **2.391E+04** $\approx$
F13	1.358E+05 ± 1.576E+05 +	**6.270E+03** ± **3.950E+03** −	1.308E+05 ± 6.233E+04 +	1.815E+05 ± 3.179E+05 +
F14	3.866E+04 ± 2.611E+04 +	5.250E+04 ± 3.640E+04 +	1.309E+07 ± 1.160E+07 +	**4.627E+03** ± **3.527E+03** −
F15	2.968E+03 ± 3.474E+02 +	2.570E+03 ± 2.840E+02 +	3.575E+03 ± 2.107E+02 +	2.668E+03 ± 2.644E+02 +
F16	2.402E+03 ± 2.503E+02 +	**2.030E+03** ± **1.550E+02** $\approx$	2.468E+03 ± 1.645E+02 +	2.190E+03 ± 1.935E+02 +
F17	1.383E+06 ± 2.697E+06 +	**1.580E+05** ± **1.380E+05** −	3.160E+06 ± 1.843E+06 +	9.911E+05 ± 1.712E+06 $\approx$
F18	5.091E+06 ± 2.563E+07 +	4.750E+05 ± 2.510E+05 +	2.688E+07 ± 1.374E+07 +	**8.001E+03** ± **6.478E+03** −
F19	2.583E+03 ± 2.296E+02 +	2.440E+03 ± 1.690E+02 $\approx$	2.617E+03 ± 1.281E+02 +	2.423E+03 ± 1.620E+02 $\approx$
F20	2.475E+03 ± 4.056E+01 +	2.400E+03 ± 4.990E+01 +	2.553E+03 ± 2.154E+01 +	**2.369E+03** ± **1.660E+01** $\approx$
F21	5.410E+03 ± 1.758E+03 +	3.510E+03 ± 1.910E+03 −	7.747E+03 ± 2.693E+03 +	**2.626E+03** ± **8.997E+02** −
F22	2.820E+03 ± 3.172E+01 +	2.760E+03 ± 3.320E+01 +	2.994E+03 ± 2.626E+01 +	2.736E+03 ± 2.115E+01 +
F23	2.971E+03 ± 3.404E+01 +	2.920E+03 ± 3.210E+01 +	3.158E+03 ± 3.168E+01 +	2.916E+03 ± 2.736E+01 +
F24	3.153E+03 ± 2.752E+02 +	2.910E+03 ± 2.270E+01 +	3.211E+03 ± 7.388E+01 +	2.897E+03 ± 1.448E+01 +
F25	5.460E+03 ± 4.864E+02 +	4.370E+03 ± 9.620E+02 +	6.947E+03 ± 2.710E+02 +	4.659E+03 ± 2.577E+02 +
F26	3.235E+03 ± 1.816E+01 +	3.230E+03 ± 1.850E+01 +	3.407E+03 ± 4.746E+01 +	3.238E+03 ± 1.302E+01 +
F27	4.073E+03 ± 8.395E+02 +	**3.220E+03** ± **2.910E+01** −	3.825E+03 ± 1.262E+02 +	3.243E+03 ± 2.597E+01 $\approx$
F28	4.021E+03 ± 2.939E+02 +	3.910E+03 ± 2.130E+02 +	4.649E+03 ± 2.115E+02 +	3.778E+03 ± 2.167E+02 +
F29	8.486E+05 ± 3.906E+06 +	1.910E+06 ± 1.150E+06 +	7.727E+07 ± 2.985E+07 +	5.730E+04 ± 3.864E+04 +
*W*/*T*/*L*	29/0/0	20/3/6	29/0/0	16/7/6

Significance values are given in Bold.

**Table 4:** The detailed *p*-values for Wilcoxon rank-sum test in 30 dimensions.

MCSMA vs	SMA	HHO	WOA	MFO	SSA	SCA	GLPSO
	*p*-value	*p*-value	*p*-value	*p*-value	*p*-value	*p*-value	*p*-value
F1	8.005E−01 $\approx$	1.652E−18 +	1.652E−18 +	7.542E−18 +	9.994E−01 −	1.652E−18 +	9.993E−01 −
F2	1.000E+00 −	1.652E−18 +	1.652E−18 +	1.652E−18 +	1.000E+00 −	1.652E−18 +	1.652E−18 +
F3	1.000E+00 −	9.916E−05 +	1.440E−11 +	3.960E−17 +	8.638E−01 $\approx$	1.652E−18 +	7.286E−03 +
F4	2.575E−03 +	1.652E−18 +	1.652E−18 +	2.217E−18 +	1.493E−13 +	1.652E−18 +	8.079E−01 $\approx$
F5	1.000E+00 −	1.652E−18 +	1.652E−18 +	1.652E−18 +	1.652E−18 +	1.652E−18 +	1.000E+00 −
F6	7.561E−03 +	1.652E−18 +	1.652E−18 +	1.421E−17 +	1.672E−15 +	1.652E−18 +	6.652E−09 +
F7	1.407E−02 +	2.217E−18 +	1.652E−18 +	2.217E−18 +	1.891E−11 +	1.652E−18 +	9.906E−01 −
F8	3.984E−09 +	2.644E−18 +	4.473E−18 +	1.267E−17 +	6.986E−15 +	2.003E−17 +	2.155E−04 +
F9	4.848E−02 +	8.768E−16 +	6.717E−18 +	2.974E−14 +	6.195E−13 +	1.652E−18 +	9.107E−01 $\approx$
F10	4.815E−07 +	2.572E−13 +	1.752E−18 +	1.652E−18 +	1.148E−15 +	1.652E−18 +	9.526E−14 +
F11	1.764E−15 +	1.652E−18 +	1.652E−18 +	1.752E−18 +	2.091E−18 +	1.652E−18 +	2.826E−14 +
F12	6.520E−02 $\approx$	1.652E−18 +	3.006E−15 +	5.060E−11 +	6.502E−13 +	1.652E−18 +	2.847E−01 $\approx$
F13	9.119E−12 +	1.571E−05 +	2.351E−18 +	6.594E−11 +	1.000E+00 −	1.652E−18 +	4.614E−03 +
F14	2.045E−03 +	5.516E−07 +	8.962E−11 +	5.902E−07 +	2.782E−09 +	1.652E−18 +	1.000E+00 −
F15	4.520E−02 +	1.606E−16 +	1.752E−18 +	5.475E−14 +	2.164E−05 +	1.652E−18 +	2.364E−08 +
F16	3.291E−03 +	9.557E−15 +	1.002E−13 +	1.891E−11 +	6.260E−01 $\approx$	1.279E−15 +	2.568E−04 +
F17	7.422E−03 +	6.799E−06 +	8.617E−14 +	2.936E−06 +	9.966E−01 −	3.152E−18 +	3.690E−01 $\approx$
F18	2.164E−05 +	5.969E−15 +	1.652E−18 +	6.123E−10 +	3.157E−17 +	1.652E−18 +	9.993E−01 −
F19	6.336E−01 $\approx$	1.146E−08 +	5.180E−10 +	4.296E−04 +	4.920E−01 $\approx$	2.426E−06 +	6.707E−01 $\approx$
F20	1.759E−06 +	1.652E−18 +	1.652E−18 +	1.505E−17 +	7.522E−11 +	1.652E−18 +	5.952E−01 $\approx$
F21	2.052E−01 $\approx$	1.359E−04 +	9.664E−10 +	1.066E−02 +	1.000E+00 −	1.984E−05 +	1.000E+00 −
F22	4.981E−07 +	1.858E−18 +	1.652E−18 +	1.752E−18 +	1.517E−09 +	1.652E−18 +	3.227E−05 +
F23	5.902E−07 +	1.652E−18 +	1.652E−18 +	4.824E−16 +	8.228E−05 +	1.652E−18 +	1.395E−04 +
F24	1.000E+00 −	4.146E−09 +	1.652E−18 +	3.342E−18 +	1.387E−03 +	1.652E−18 +	9.119E−12 +
F25	1.587E−04 +	1.002E−13 +	3.670E−16 +	3.960E−17 +	1.011E−02 +	1.652E−18 +	4.653E−07 +
F26	6.107E−01 $\approx$	1.752E−18 +	1.752E−18 +	1.046E−11 +	8.086E−09 +	1.652E−18 +	1.239E−14 +
F27	5.900E−01 $\approx$	5.479E−01 $\approx$	1.524E−14 +	2.091E−18 +	9.998E−01 −	1.652E−18 +	1.319E−01 $\approx$
F28	3.320E−05 +	7.781E−17 +	1.752E−18 +	3.626E−13 +	3.780E−12 +	1.652E−18 +	8.159E−06 +
F29	6.985E−07 +	1.652E−18 +	1.652E−18 +	5.556E−17 +	1.652E−18 +	1.652E−18 +	1.088E−16 +
*W*/*T*/*L*	19/6/4	28/1/0	29/0/0	29/0/0	20/3/6	29/0/0	16/7/6

**Table 5:** The CEC2017 experimental results of MCSMA and other comparative algorithms in 50 dimensions.

	MCSMA	SMA	HHO	WOA
	MEAN STD	MEAN STD	MEAN STD	MEAN STD
F1	6.525E+03 ± 7.346E+03	1.347E+04 ± 1.127E+04 +	4.057E+07 ± 6.459E+06 +	3.577E+07 ± 1.860E+07 +
F2	**3.204E+02** ± **1.079E+01**	3.300E+02 ± 1.330E+01 +	9.566E+03 ± 2.580E+03 +	9.805E+04 ± 4.776E+04 +
F3	**5.620E+02** ± **4.545E+01**	5.729E+02 ± 3.817E+01 $\approx$	6.452E+02 ± 5.030E+01 +	7.215E+02 ± 5.938E+01 +
F4	6.758E+02 ± 3.966E+01	6.950E+02 ± 3.680E+01 +	8.731E+02 ± 3.490E+01 +	9.177E+02 ± 8.521E+01 +
F5	6.093E+02 ± 2.031E+00	6.057E+02 ± 4.717E+00 −	6.700E+02 ± 5.483E+00 +	6.788E+02 ± 1.020E+01 +
F6	9.465E+02 ± 3.848E+01	9.713E+02 ± 4.445E+01 +	1.768E+03 ± 6.871E+01 +	1.680E+03 ± 1.068E+02 +
F7	9.674E+02 ± 3.187E+01	9.838E+02 ± 3.672E+01 +	1.160E+03 ± 2.768E+01 +	1.243E+03 ± 7.746E+01 +
F8	4.753E+03 ± 2.852E+03	9.780E+03 ± 4.310E+03 +	1.740E+04 ± 2.133E+03 +	2.288E+04 ± 6.552E+03 +
F9	6.638E+03 ± 8.346E+02	6.737E+03 ± 8.142E+02 $\approx$	8.547E+03 ± 9.235E+02 +	1.029E+04 ± 1.292E+03 +
F10	**1.279E+03** ± **5.001E+01**	1.342E+03 ± 7.082E+01 +	1.439E+03 ± 8.570E+01 +	1.691E+03 ± 1.311E+02 +
F11	**1.715E+06** ± **1.024E+06**	7.904E+06 ± 3.719E+06 +	5.247E+07 ± 2.266E+07 +	3.038E+08 ± 1.761E+08 +
F12	**2.496E+04** ± **1.137E+04**	3.648E+04 ± 8.919E+03 +	1.468E+06 ± 7.604E+05 +	4.003E+05 ± 3.578E+05 +
F13	**1.192E+05** ± **6.477E+04**	1.702E+05 ± 1.108E+05 +	3.642E+05 ± 2.649E+05 +	8.510E+05 ± 6.390E+05 +
F14	2.156E+04 ± 9.497E+03	2.961E+04 ± 6.439E+03 +	2.225E+05 ± 1.022E+05 +	1.218E+05 ± 9.896E+04 +
F15	**2.976E+03** ± **4.146E+02**	3.184E+03 ± 4.831E+02 +	4.190E+03 ± 5.372E+02 +	5.033E+03 ± 7.102E+02 +
F16	**2.678E+03** ± **3.228E+02**	3.077E+03 ± 3.676E+02 +	3.623E+03 ± 3.749E+02 +	4.041E+03 ± 4.696E+02 +
F17	**6.480E+05** ± **3.728E+05**	1.117E+06 ± 5.920E+05 +	2.482E+06 ± 1.471E+06 +	6.463E+06 ± 5.285E+06 +
F18	1.899E+04 ± 1.743E+04	**9.599E+03** ± **1.250E+04** $\approx$	4.896E+05 ± 3.200E+05 +	3.231E+06 ± 2.784E+06 +
F19	**2.949E+03** ± **2.854E+02**	2.962E+03 ± 2.839E+02 $\approx$	3.419E+03 ± 3.164E+02 +	3.554E+03 ± 3.206E+02 +
F20	2.467E+03 ± 3.445E+01	2.494E+03 ± 3.787E+01 +	2.805E+03 ± 7.453E+01 +	2.863E+03 ± 9.134E+01 +
F21	8.475E+03 ± 9.895E+02	8.266E+03 ± 9.897E+02 $\approx$	1.072E+04 ± 9.518E+02 +	1.193E+04 ± 1.364E+03 +
F22	**2.892E+03** ± **3.273E+01**	2.943E+03 ± 4.086E+01 +	3.601E+03 ± 1.276E+02 +	3.601E+03 ± 1.535E+02 +
F23	**3.058E+03** ± **3.900E+01**	3.111E+03 ± 4.684E+01 +	4.107E+03 ± 1.326E+02 +	3.671E+03 ± 1.816E+02 +
F24	3.058E+03 ± 2.958E+01	3.040E+03 ± 2.965E+01 −	3.130E+03 ± 3.774E+01 +	3.178E+03 ± 5.223E+01 +
F25	**4.229E+03** ± **1.337E+03**	5.238E+03 ± 1.324E+03 +	9.660E+03 ± 2.707E+03 +	1.298E+04 ± 1.579E+03 +
F26	**3.342E+03** ± **6.650E+01**	3.375E+03 ± 7.489E+01 +	3.991E+03 ± 2.478E+02 +	4.320E+03 ± 3.928E+02 +
F27	3.313E+03 ± 2.418E+01	3.299E+03 ± 2.293E+01 −	3.357E+03 ± 3.746E+01 +	3.517E+03 ± 9.187E+01 +
F28	**3.984E+03** ± **2.953E+02**	4.195E+03 ± 2.575E+02 +	5.320E+03 ± 5.296E+02 +	7.232E+03 ± 7.154E+02 +
F29	**1.414E+06** ± **4.021E+05**	1.637E+06 ± 3.666E+05 +	1.474E+07 ± 3.283E+06 +	1.113E+08 ± 4.663E+07 +

*W*/*T*/*L*	${-}{-}/{-}{-}/{-}{-}$	21/5/3	29/0/0	29/0/0
	MFO	SSA	SCA	GLPSO
	MEAN STD	MEAN STD	MEAN STD	MEAN STD
F1	3.362E+10 ± 1.444E+10 +	6.571E+03 ± 8.682E+03 $\approx$	5.099E+10 ± 5.797E+09 +	**4.108E+04** ± **1.725E+05** $\approx$
F2	1.776E+05 ± 7.814E+04 +	1.386E+03 ± 1.028E+03 +	1.383E+05 ± 1.953E+04 +	6.296E+04 ± 3.565E+04 +
F3	3.283E+03 ± 1.857E+03 +	5.685E+02 ± 4.637E+01 $\approx$	8.073E+03 ± 1.691E+03 +	5.671E+02 ± 4.378E+01 +
F4	9.253E+02 ± 8.262E+01 +	7.966E+02 ± 8.042E+01 +	1.100E+03 ± 3.605E+01 +	**6.450E+02** ± **3.219E+01** −
F5	6.475E+02 ± 8.346E+00 +	6.490E+02 ± 9.830E+00 +	6.756E+02 ± 5.819E+00 +	**6.002E+02** ± **5.951E-02** −
F6	1.649E+03 ± 3.968E+02 +	1.041E+03 ± 7.306E+01 +	1.730E+03 ± 7.505E+01 +	**9.596E+02** ± **4.366E+01** $\approx$
F7	1.214E+03 ± 6.741E+01 +	1.095E+03 ± 6.373E+01 +	1.409E+03 ± 3.165E+01 +	**9.382E+02** ± **2.864E+01** −
F8	1.548E+04 ± 4.078E+03 +	1.085E+04 ± 2.664E+03 +	2.695E+04 ± 5.018E+03 +	**2.228E+03** ± **8.533E+02** −
F9	8.495E+03 ± 1.112E+03 +	7.664E+03 ± 7.800E+02 +	1.461E+04 ± 3.521E+02 +	**5.651E+03** ± **6.510E+02** −
F10	7.392E+03 ± 5.936E+03 +	1.399E+03 ± 7.994E+01 +	8.584E+03 ± 1.670E+03 +	3.935E+03 ± 2.659E+03 +
F11	4.015E+09 ± 3.442E+09 +	2.659E+07 ± 1.821E+07 +	1.480E+10 ± 2.850E+09 +	8.969E+06 ± 6.580E+06 +
F12	3.489E+08 ± 6.208E+08 +	1.292E+05 ± 9.021E+04 +	4.031E+09 ± 1.247E+09 +	4.824E+04 ± 8.263E+04 $\approx$
F13	1.071E+06 ± 1.399E+06 +	7.723E+04 ± 6.164E+04 −	2.760E+06 ± 1.217E+06 +	2.069E+06 ± 2.430E+06 +
F14	2.766E+07 ± 8.386E+07 +	5.677E+04 ± 4.327E+04 +	6.767E+08 ± 2.486E+08 +	**1.723E+04** ± **3.284E+04** −
F15	4.118E+03 ± 5.162E+02 +	3.342E+03 ± 4.671E+02 +	5.748E+03 ± 3.818E+02 +	3.458E+03 ± 4.214E+02 +
F16	3.931E+03 ± 4.015E+02 +	3.184E+03 ± 3.617E+02 +	4.664E+03 ± 3.071E+02 +	2.925E+03 ± 2.848E+02 +
F17	5.603E+06 ± 8.669E+06 +	6.282E+05 ± 4.406E+05 $\approx$	2.184E+07 ± 9.563E+06 +	5.992E+06 ± 5.756E+06 +
F18	6.376E+06 ± 2.521E+07 +	1.202E+06 ± 6.002E+05 +	3.975E+08 ± 1.590E+08 +	1.567E+04 ± 8.471E+03 $\approx$
F19	3.485E+03 ± 3.327E+02 +	3.062E+03 ± 2.806E+02 +	4.003E+03 ± 1.587E+02 +	2.972E+03 ± 3.482E+02 $\approx$
F20	2.720E+03 ± 7.047E+01 +	2.550E+03 ± 6.317E+01 +	2.907E+03 ± 3.667E+01 +	**2.447E+03** ± **3.435E+01** −
F21	1.020E+04 ± 1.024E+03 +	9.176E+03 ± 1.558E+03 +	1.622E+04 ± 3.493E+02 +	**7.433E+03** ± **7.999E+02** −
F22	3.126E+03 ± 6.074E+01 +	2.981E+03 ± 7.560E+01 +	3.524E+03 ± 5.300E+01 +	2.913E+03 ± 4.147E+01 +
F23	3.205E+03 ± 5.237E+01 +	3.113E+03 ± 5.402E+01 +	3.697E+03 ± 5.206E+01 +	3.154E+03 ± 7.540E+01 +
F24	5.479E+03 ± 2.029E+03 +	**3.037E+03** ± **2.643E+01** −	7.177E+03 ± 8.641E+02 +	3.071E+03 ± 2.633E+01 +
F25	7.883E+03 ± 6.401E+02 +	4.566E+03 ± 2.008E+03 $\approx$	1.206E+04 ± 7.289E+02 +	5.685E+03 ± 4.049E+02 +
F26	3.554E+03 ± 6.808E+01 +	3.455E+03 ± 9.541E+01 +	4.364E+03 ± 1.755E+02 +	3.454E+03 ± 6.385E+01 +
F27	7.948E+03 ± 1.106E+03 +	**3.294E+03** ± **2.508E+01** −	7.252E+03 ± 7.618E+02 +	3.336E+03 ± 3.640E+01 +
F28	5.151E+03 ± 5.115E+02 +	4.911E+03 ± 3.634E+02 +	7.558E+03 ± 7.231E+02 +	4.160E+03 ± 2.748E+02 +
F29	4.582E+07 ± 1.036E+08 +	4.263E+07 ± 8.367E+06 +	9.126+08 ± 2.718E+08 +	1.132E+06 ± 2.228E+05 $\approx$
*W*/*T*/*L*	29/0/0	22/4/3	29/0/0	15/6/8

Significance values are given in Bold.

**Table 6:** The CEC2017 experimental results of MCSMA and other comparative algorithms in 100 dimensions.

	MCSMA	SMA	HHO	WOA
	MEAN STD	MEAN STD	MEAN STD	MEAN STD
F1	1.447E+04 ± 1.524E+04	4.258E+05 ± 2.977E+05 +	3.283E+08 ± 3.882E+07 +	1.234E+09 ± 4.214E+08 +
F2	3.802E+04 ± 1.119E+04	**2.010E+04** ± **7.343E+03** −	1.343E+05 ± 1.835E+04 +	7.631E+05 ± 2.247E+05 +
F3	7.284E+02 ± 5.529E+01	**6.765E+02** ± **4.166E+01** −	9.829E+02 ± 8.617E+01 +	1.506E+03 ± 1.797E+02 +
F4	**1.001E+03** ± **7.673E+01**	1.096E+03 ± 9.595E+01 +	1.441E+03 ± 6.315E+01 +	1.497E+03 ± 1.076E+02 +
F5	6.327E+02 ± 8.062E+00	6.265E+02 ± 6.964E+00 −	6.802E+02 ± 3.314E+00 +	6.845E+02 ± 9.276E+00 +
F6	1.447E+03 ± 1.082E+02	1.526E+03 ± 1.244E+02 +	3.593E+03 ± 1.231E+02 +	3.301E+03 ± 1.487E+02 +
F7	**1.274E+03** ± **7.186E+01**	1.368E+03 ± 9.516E+01 +	1.901E+03 ± 7.126E+01 +	1.942E+03 ± 1.155E+02 +
F8	2.225E+04 ± 3.272E+03	2.549E+04 ± 3.849E+03 +	3.949E+04 ± 4.424E+03 +	4.956E+04 ± 1.292E+04 +
F9	1.465E+04 ± 1.275E+03	**1.388E+04** ± **1.157E+03** −	2.016E+04 ± 1.710E+03 +	2.199E+04 ± 2.237E+03 +
F10	**2.282E+03** ± **1.859E+02**	2.260E+03 ± 1.936E+02 $\approx$	3.376E+03 ± 3.343E+02 +	4.228E+04 ± 2.780E+04 +
F11	**8.918E+06** ± **4.553E+06**	3.746E+07 ± 1.605E+07 +	4.258E+08 ± 1.370E+08 +	1.207E+09 ± 4.695E+08 +
F12	**1.613E+04** ± **1.144E+04**	6.552E+04 ± 2.336E+04 +	5.164E+06 ± 1.090E+06 +	9.358E+05 ± 7.259E+05 +
F13	**4.773E+05** ± **2.863E+05**	1.352E+06 ± 6.596E+05 +	1.480E+06 ± 6.066E+05 +	4.099E+06 ± 1.901E+06 +
F14	1.203E+04 ± 9.271E+03	2.493E+04 ± 1.307E+04 +	1.718E+06 ± 2.304E+06 +	5.632E+05 ± 2.852E+06 +
F15	**5.060E+03** ± **6.968E+02**	5.486E+03 ± 5.878E+02 +	7.353E+03 ± 9.509E+02 +	1.192E+04 ± 1.896E+03 +
F16	**4.830E+03** ± **4.869E+02**	5.014E+03 ± 5.279E+02 +	6.254E+03 ± 6.681E+02 +	7.276E+03 ± 8.013E+02 +
F17	1.464E+06 ± 7.238E+05	2.968E+06 ± 1.534E+06 +	2.810E+06 ± 9.476E+05 +	4.140E+06 ± 2.209E+06 +
F18	1.856E+04 ± 1.363E+04	2.367E+04 ± 1.373E+04 +	5.482E+06 ± 2.347E+06 +	2.344E+07 ± 1.350E+07 +
F19	**4.817E+03** ± **5.785E+02**	4.951E+03 ± 5.066E+02 $\approx$	5.864E+03 ± 4.342E+02 +	6.261E+03 ± 6.180E+02 +
F20	**2.822E+03** ± **6.455E+01**	2.895E+03 ± 8.069E+01 +	3.860E+03 ± 1.422E+02 +	3.983E+03 ± 1.880E+02 +
F21	**1.665E+04** ± **1.441E+03**	1.699E+04 ± 1.432E+03 $\approx$	2.321E+04 ± 1.361E+03 +	2.530E+04 ± 2.405E+03 +
F22	**3.183E+03** ± **5.300E+01**	3.244E+03 ± 7.330E+01 +	4.772E+03 ± 2.329E+02 +	4.773E+03 ± 2.646E+02 +
F23	**3.748E+03** ± **7.860E+01**	3.828E+03 ± 9.165E+01 +	6.085E+03 ± 3.581E+02 +	6.118E+03 ± 3.612E+02 +
F24	3.401E+03 ± 5.873E+01	**3.324E+03** ± **6.035E+01** −	3.649E+03 ± 8.107E+01 +	4.035E+03 ± 1.410E+02 +
F25	**1.032E+04** ± **1.651E+03**	1.155E+04 ± 8.403E+02 +	2.550E+04 ± 1.912E+03 +	3.155E+04 ± 3.254E+03 +
F26	**3.461E+03** ± **4.328E+01**	3.479E+03 ± 5.525E+01 +	4.344E+03 ± 2.679E+02 +	4.950E+03 ± 5.799E+02 +
F27	3.491E+03 ± 4.220E+01	**3.421E+03** ± **4.478E+01** −	3.642E+03 ± 5.566E+01 +	4.368E+03 ± 1.965E+02 +
F28	**6.299E+03** ± **5.196E+02**	6.591E+03 ± 6.012E+02 +	9.293E+03 ± 8.715E+02 +	1.437E+04 ± 1.757E+03 +
F29	**1.185E+04** ± **5.198E+03**	1.044E+05 ± 3.904E+04 +	3.448E+07 ± 1.036E+07 +	3.933E+08 ± 1.679E+08 +

*W*/*T*/*L*	${-}{-}/{-}{-}/{-}{-}$	20/3/6	29/0/0	29/0/0
	MFO	SSA	SCA	GLPSO
	MEAN STD	MEAN STD	MEAN STD	MEAN STD
F1	1.317E+11 ± 4.122E+10 +	**1.029E+04** ± **1.217E+04** −	2.921E+11 ± 3.109E+10 +	2.772E+09 ± 7.348E+08 +
F2	6.593E+05 ± 1.489E+05 +	1.362E+05 ± 2.476E+04 +	6.796E+05 ± 6.356E+04 +	4.696E+05 ± 4.644E+04 +
F3	2.121E+04 ± 1.061E+04 +	6.999E+02 ± 4.960E+01 −	8.244E+04 ± 1.686E+04 +	1.470E+03 ± 1.976E+02 +
F4	1.694E+03 ± 1.553E+02 +	1.253E+03 ± 1.109E+02 +	2.333E+03 ± 8.828E+01 +	1.000E+03 ± 7.633E+01 +
F5	6.678E+02 ± 6.332E+00 +	6.614E+02 ± 6.009E+00 +	7.221E+02 ± 7.726E+00 +	**6.063E+02** ± **1.908E+00** −
F6	3.978E+03 ± 7.224E+02 +	1.748E+03 ± 1.657E+02 +	6.664E+03 ± 1.648E+03 +	1.525E+03 ± 1.265E+02 +
F7	2.025E+03 ± 1.338E+02 +	1.581E+03 ± 1.224E+02 +	2.678E+03 ± 8.331E+01 +	1.330E+03 ± 5.685E+01 +
F8	4.300E+04 ± 4.461E+03 +	2.460E+04 ± 2.782E+03 +	1.374E+05 ± 1.414E+04 +	**4.871E+03** ± **2.337E+03** −
F9	1.738E+04 ± 2.027E+03 +	1.564E+04 ± 1.497E+03 +	3.210E+04 ± 4.956E+02 +	3.024E+04 ± 5.417E+02 +
F10	1.357E+05 ± 6.761E+04 +	2.852E+03 ± 2.326E+02 +	2.172E+05 ± 3.358E+04 +	1.021E+05 ± 4.352E+04 +
F11	3.219E+10 ± 1.479E+10 +	2.427E+08 ± 1.138E+08 +	1.130E+11 ± 1.269E+10 +	3.217E+08 ± 9.073E+07 +
F12	3.962E+09 ± 3.015E+09 +	8.150E+04 ± 3.745E+04 +	2.014E+10 ± 3.297E+09 +	1.192E+04 ± 5.917E+03 +
F13	9.816E+06 ± 1.458E+07 +	5.991E+05 ± 3.777E+05 $\approx$	7.558E+07 ± 2.239E+07 +	3.398E+06 ± 2.422E+06 +
F14	8.167E+08 ± 9.519E+08 +	7.385E+04 ± 3.036E+04 +	7.214E+09 ± 1.522E+09 +	**5.291E+03** ± **3.355E+03** $\approx$
F15	7.997E+03 ± 9.140E+02 +	6.167E+03 ± 7.352E+02 +	1.512E+04 ± 7.345E+02 +	5.668E+03 ± 6.391E+02 +
F16	7.937E+03 ± 1.673E+03 +	5.155E+03 ± 5.879E+02 +	4.236E+04 ± 2.817E+04 +	5.028E+03 ± 6.261E+02 +
F17	1.253E+07 ± 1.499E+07 +	**1.164E+06** ± **5.757E+05** −	1.541E+08 ± 4.782E+07 +	3.162E+06 ± 2.456E+06 +
F18	7.058E+08 ± 7.901E+08 +	5.300E+06 ± 2.009E+06 +	6.437E+09 ± 1.267E+09 +	**6.272E+03** ± **4.356E+03** $\approx$
F19	5.700E+03 ± 5.766E+02 +	5.062E+03 ± 5.885E+02 +	7.843E+03 ± 2.190E+02 +	6.964E+03 ± 2.102E+02 +
F20	3.566E+03 ± 1.366E+02 +	3.041E+03 ± 1.311E+02 +	4.279E+03 ± 9.810E+01 +	2.916E+03 ± 9.930E+01 +
F21	1.989E+04 ± 1.757E+03 +	1.812E+04 ± 1.490E+03 +	3.410E+04 ± 5.073E+02 +	3.176E+04 ± 5.610E+03 +
F22	3.724E+03 ± 9.394E+01 +	3.469E+03 ± 1.170E+02 +	4.908E+03 ± 8.796E+01 +	3.433E+03 ± 7.813E+01 +
F23	4.270E+03 ± 1.343E+02 +	3.969E+03 ± 1.292E+02 +	6.528E+03 ± 1.857E+02 +	4.116E+03 ± 9.279E+01 +
F24	1.251E+04 ± 5.666E+03 +	3.352E+03 ± 6.509E+01 −	4.610E+04 ± 8.525E+03 +	4.073E+03 ± 1.714E+02 +
F25	1.684E+04 ± 1.257E+03 +	1.233E+04 ± 3.947E+03 +	3.773E+04 ± 1.917E+03 +	1.448E+04 ± 1.407E+03 +
F26	3.890E+03 ± 1.755E+02 +	3.683E+03 ± 8.215E+01 +	7.414E+03 ± 3.846E+02 +	3.873E+03 ± 7.320E+01 +
F27	1.793E+04 ± 2.278E+03 +	3.451E+03 ± 4.782E+01 −	3.742E+04 ± 2.995E+03 +	4.183E+03 ± 1.611E+02 +
F28	1.099E+04 ± 5.414E+03 +	8.573E+03 ± 7.588E+02 +	4.727E+04 ± 1.849E+04 +	6.894E+03 ± 5.809E+02 +
F29	1.875E+09 ± 1.311E+09 +	4.359E+07 ± 2.236E+07 +	1.285E+10 ± 1.661E+09 +	3.932E+05 ± 1.893E+05 +
*W*/*T*/*L*	29/0/0	23/1/5	29/0/0	25/2/2

Significance values are given in Bold.

### Engineering practical problem test

In the past, the research of intelligent algorithms mainly focused on mathematical theory and simulation modeling. With the rising productivity needs of society, whether an algorithm is superior should also focus on its ability to solve real engineering problems and create social value^54–56^. To test the capability of MCSMA on some large-scale complex real-world problems, we choose CEC2011 as a test set. CEC2011 contains 22 test functions, encompassing real-world problems in various domains. Table 7 reveals the details of these real-world problems, including dimensions, constraint types, and modeling processes.

**Table 7:** The summary of CEC2011 test problems.

Problem No.	Dimensions	Constraints	Definitions of problems
F1	6	Bounded	To solve the multimodal problem in the music field regarding optimizing parameters for FM sound waves to simulate timbres.
F2	30	Bounded	To test the algorithm’s performance in tuning the structure of Lennard-Jones, a 20-sided body, so that the molecular potential energy is minimized.
F3	1	Bouned	A chemical catalyst conversion methyl optimisation problem dealing with multiple systems of differential equations simultaneously.
F4	1	Unconstrained	The problem of optimal control of chemical reactions in stirred reactors, finding the most suitable value for the coolant flow rate to maximize the target chemical product.
F5	30	Bounded	To address the problem of minimizing the potential energy between silicon atoms in covalent power systems.
F6	30	Bounded	The problem of selecting the appropriate waveform to handle the radar pulse is formulated as a non-linear optimization problem with continuous variables.
F7	20	Bounded	The optimization problem of planning a collection of transmission lines to minimize their cost while meeting constraints on line power.
F8	7	Equality and inequality	Solving the pricing problems in the power system which is a multi-factorial constrained optimization concerning costs, benefits, loss rates, markets, etc.
F9	126	Linear equality	To design an antenna array, considering its received signal area, orientation angle, and the proper arrangement between array patterns.
F10	12	Bounded	The problem of non-reciprocal constraints in dynamically dispatching generation schedules based on each hour’s demand for electricity.
F11	120	Inequality	Aimed at statically dispatching the fuel cost optimization problem of a generating unit within an operating cycle. Seven problems are designed based on upper and lower limits of operating power and conflicting factors between specific regions. This complex problem can be classified as a multimodal multi-constraint optimization problem under different dimensional conditions.
F12	216		
F13	6		
F14	13		
F15	15		
F16	40		
F17	140		
F18-F20	96	Inequality	The problem of rationalizing the integration of hydroelectric and thermal power generation in a hydrothermal power system to meet load requirements. It is designed as 3 problems taking into account fuel cost, hydro network, delayed scheduling, etc.
F21	26	Bounded	Orbit planning for spacecraft under multiple gravity fields with propulsion engines, which is a large-scale global optimization problem.
F22	22	Bounded	A high-dimensional continuous optimization problem of multiple interplanetary spacecraft orbits.

Table 8 shows the comparison results between MCSMA and the other six meta-heuristic algorithms. The comparison result of “*W*/*T*/*L*” with the original SMA is 8/8/6. Although MCSMA does not achieve a significant advantage, it obtains the most number of optimal values, indicating that the method we proposed for improvement still has some performance boost in solving complex real-world problems. Compared with other meta-heuristic algorithms, MCSMA shows considerably satisfactory comparison results. In particular, MCSMA achieves the most optimal values for some multimodal high-dimensional test problems, indicating that MCSMA has considerable potential and advantages for some complex application problems. Therefore, MCSMA can be applied to the present engineering and practical fields with desirable results tentatively in the future^57,58^.

**Table 8:** Results of MCSMA and other heuristic algorithms on the CEC2011 real-world problem.

	MCSMA	SMA	HHO	WOA	MFO	SSA	SCA
	MEAN STD	MEAN STD	MEAN STD	MEAN STD	MEAN STD	MEAN STD	MEAN STD
F1	**1.233E+01** ± **4.976E+00**	1.611E+01 ± 6.061E+00 +	1.531E+01 ± 4.399E+00 +	1.728E+01 ± 6.049E+00 +	1.743E+01 ± 5.064E+00 +	1.528E+01 ± 6.050E+00 +	1.376E+01 ± 3.220E+00 $\approx$
F2	− 2.003E+01 ± 5.176E+00	**− 1.604E+01** ± **5.135E+00** +	−2.176E+01 ± 2.471E+00 $\approx$	−1.995E+01 ± 4.015E+00 $\approx$	−1.269E+01 ± 3.678E+00 +	−1.949E+01 ± 3.163E+00 $\approx$	−1.023E+01 ± 1.539E+00 +
F3	1.151E−05 ± 4.991E−15	1.152E−05 ± 1.323E−09 +	**1.151E−05** ± **4.528E−19** −	1.151E−05 ± 1.262E−17 −	1.151E−05 ± 2.944E−19 −	1.151E−05 ± 3.793E−18 −	1.151E−05 ± 9.473E−12 +
F4	1.425E+01 ± 1.771E−01	1.420E+01 ± 2.349E−01 −	1.423E+01 ± 2.275E−01 −	**1.423E+01** ± **2.063E−01** −	1.771E+01 ± 3.426E+00 $\approx$	1.483E+01 ± 1.669E+00 −	1.532E+01 ± 1.517E+00 +
F5	−3.286E+01 ± 1.527E+00	**− 3.285E+01** ± **2.023E+00** −	− 2.665E+01 ± 2.840E+00 +	−2.686E+01 ± 4.104E+00 +	−3.094E+01 ± 4.084E+00 $\approx$	−2.845E+01 ± 3.244E+00 +	−2.036E+01 ± 1.827E+00 +
F6	−2.519E+01 ± 3.361E+00	**− 2.563E+01** ± **3.469E+00** −	− 2.120E+01 ± 2.934E+00 +	−1.965E+01 ± 3.419E+00 +	−2.503E+01 ± 3.564E+00 $\approx$	−2.040E+01 ± 3.750E+00 +	−1.441E+01 ± 1.470E+00 +
F7	1.525E+00 ± 2.255E−01	**9.714E−01** ± **1.863E−01** −	1.590E+00 ± 1.919E−01 $\approx$	1.764E+00 ± 2.115E−01 +	1.236E+00 ± 2.264E−01 −	1.029E+00 ± 1.752E−01 −	1.933E+00 ± 2.073E−01 +
F8	**2.200E+02** ± **0.000E+00**	2.200E+02 ± 0.000E+00 $\approx$	2.354E+02 ± 2.931E+01 +	2.698E+02 ± 2.798E+01 +	2.372E+02 ± 2.119E+01 +	2.476E+02 ± 3.116E+01 +	3.742E+02 ± 2.218E+02 +
F9	**3.335E+03** ± **1.750E+03**	4.054E+03 ± 1.149E+03 +	3.534E+05 ± 1.096E+05 +	2.840E+05 ± 1.164E+05 +	3.828E+04 ± 3.806E+04 +	1.449E+04 ± 1.098E+04 +	3.911E+05 ± 8.225E+04 +
F10	**− 1.778E+01** ± **3.052E+00**	−1.403E+01 ± 3.002E+00 +	−1.150E+01 ± 6.056E−01 +	−1.071E+01 ± 1.403E+00 +	−1.269E+01 ± 1.492E+00 +	−1.256E+01 ± 2.414E+00 +	−1.074E+01 ± 5.344E−01 +
F11	5.249E+04 ± 6.074E+02	**5.228E+04** ± **5.297E+02** $\approx$	8.274E+04 ± 4.819E+03 +	1.030E+06 ± 1.737E+05 +	9.582E+06 ± 9.528E+06 +	6.275E+04 ± 1.773E+04 +	1.739E+08 ± 1.805E+07 +
F12	**1.734E+07** ± **2.108E+04**	1.738E+07 ± 3.536E+04 +	1.821E+07 ± 1.309E+05 +	2.376E+07 ± 1.110E+06 +	2.386E+07 ± 1.683E+06 +	1.874E+07 ± 2.565E+05 +	4.670E+07 ± 9.229E+05 +
F13	**1.548E+04** ± **3.082E+01**	1.551E+04 ± 5.418E+01 $\approx$	1.551E+04 ± 6.582E+01 +	1.554E+04 ± 7.134E+01 +	1.552E+04 ± 3.303E+01 +	1.551E+04 ± 3.449E+01 +	1.566E+04 ± 1.492E+02 +
F14	**1.914E+04** ± **1.342E+02**	1.914E+04 ± 2.137E+02 $\approx$	1.934E+04 ± 1.954E+02 +	1.934E+04 ± 2.179E+02 +	1.925E+04 ± 2.038E+02 $\approx$	1.889E+04 ± 1.487E+02 −	1.939E+04 ± 6.335E+02 $\approx$
F15	**3.288E+04** ± **8.071E+01**	3.298E+04 ± 1.317E+02 +	3.322E+04 ± 9.686E+01 +	4.886E+04 ± 7.116E+04 +	3.303E+04 ± 8.867E+01 +	3.334E+04 ± 1.209E+03 +	6.219E+04 ± 3.344E+04 +
F16	**1.319E+05** ± **2.870E+03**	1.340E+05 ± 3.224E+03 +	1.478E+05 ± 5.854E+03 +	1.478E+05 ± 7.415E+03 +	1.422E+05 ± 5.981E+03 +	1.429E+05 ± 8.169E+03 +	2.102E+05 ± 9.053E+04 +
F17	1.952E+06 ± 2.261E+04	**1.941E+06** ± **2.216E+04** −	2.734E+06 ± 6.822E+05 +	1.099E+10 ± 3.175E+09 +	2.745E+08 ± 4.263E+08 +	2.156E+06 ± 3.356E+05 +	8.727E+09 ± 1.337E+09 +
F18	9.503E+05 ± 5.144E+03	**9.503E+05** ± **4.570E+03** $\approx$	1.437E+06 ± 4.662E+05 +	2.031E+06 ± 2.227E+06 +	1.072E+06 ± 3.240E+05 +	9.641E+05 ± 6.388E+04 $\approx$	2.360E+07 ± 4.870E+06 +
F19	**1.107E+06** ± **9.647E+04**	1.151E+06 ± 1.127E+05 $\approx$	2.112E+06 ± 8.109E+05 +	2.734E+06 ± 1.547E+06 +	1.335E+06 ± 1.994E+05 +	1.591E+06 ± 2.263E+05 +	2.321E+07 ± 4.352E+06 +
F20	**9.513E+05** ± **3.867E+03**	9.547E+05 ± 1.710E+04 $\approx$	1.506E+06 ± 4.665E+05 +	2.265E+06 ± 2.069E+06 +	1.080E+06 ± 3.773E+05 +	1.004E+06 ± 2.047E+05 $\approx$	2.327E+07 ± 4.152E+06 +
F21	1.705E+01 ± 1.693E+00	**1.561E+01** ± **2.163E+00** −	3.146E+01 ± 7.239E+00 +	3.701E+01 ± 7.379E+00 +	2.177E+01 ± 5.454E+00 +	2.748E+01 ± 4.243E+00 +	4.168E+01 ± 4.647E+00 +
F22	1.854E+01 ± 3.581E+00	**1.698E+01** ± **4.342E+00** $\approx$	3.750E+01 ± 5.885E+00 +	3.834E+01 ± 6.525E+00 +	2.421E+01 ± 4.229E+00 +	2.639E+01 ± 2.625E+00 +	3.734E+01 ± 6.260E+00 +
*W*/*T*/*L*	${-}{-}/{-}{-}/{-}{-}$	8/8/6/	18/2/2	19/1/2	16/4/2	15/3/4	20/2/0

Significance values are given in Bold.

### Performance test on artificial neural model training

Currently, neural networks have become a cornerstone technology in solving image processing and classification prediction problems. Starting from the most primitive linear threshold artificial neural network models, many new network models with innovative structures and simulations of human brain structures have emerged^59,60^. Dendritic neuron model(DNM) is a single neural network model that simulates the primitive dendritic structure of a nerve cell. This model uses logical operators and sigmoid functions to transmit signals and simulates the connections and propagation between neurons. Because of its specific synaptic hierarchy, DNM can circumvent some common defects of traditional propagation networks^61^. In this section, we use DNM to examine the feasibility and performance of MCSMA on some general classification problems.

**Figure 7:**
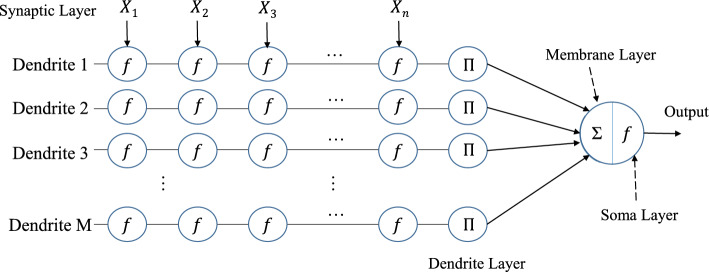
A schematic diagram of the fully connected dendritic neuron model.

The network structure of DNM is simulated from the cytosolic conformation of human brain neurons, which consists of four levels: synapse, dendrite, cell membrane, and soma. Figure 7 illustrates the general structure of a simple fully connected dendritic neural network. Similar to actual brain cells, the main role of synapses is to receive and hold messages. In DNM, the sigmoid function is selected as the activation function for each synapse. The sigmoid function protects the integrity of the data while compressing it. Thus, setting the sigmoid function as the activation function in the synaptic layer can effectively protect and process the data signal to a large extent^62^. Similar to brain potential signals, there are two states of cellular potentials depending on the input signal received by the synaptic layer: inhibition and excitation. The two states are represented and regulated by two learnable parameters in the sigmoid function.

The dendritic hierarchy is the core structure of the entire network model, where each node on each neural branch receives signals from the synapse. The process can be considered as a nonlinear mapping. According to brain science, the processing and responses produced by each response center in the cerebral cortex after receiving signals from neuronal stimuli can be regarded as a primitive multiplicative law. Between each dendritic node, multiplicative logic operations are used to prepare the signal for subsequent processing. After this, the signals from each dendritic branch are concentrated at the membrane-layer structure. At the membrane layer structure, all signals are summed linearly and the total signal is then transmitted to the cytosol for the nucleus to make the final decision. In the soma layer, a threshold exists within the cell body to determine whether the neuron emits an electrical signal. After the total potential signal processed by the first three layers exceeds the value, the neuron generates an excitation potential and transmits that excitation potential to other neural units. That is a complete single-neuron processing process^63^.

**Figure 8:**
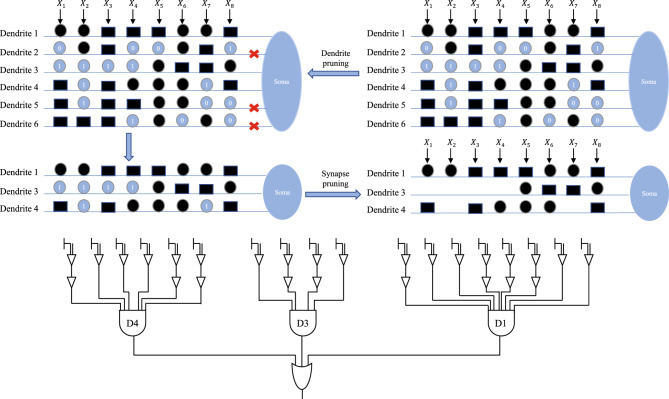
The general learning procedure of dendritic neural network model.

Potential signals in the dendritic layer will be selectively pruned according to the hierarchical structure and functional properties of DNM. When the output of any nerve node is 0, that dendritic nerve is considered an invalid dendrite to retain the robust unit with the strongest impact on the soma body. Due to the electrical principle of the system, the operation of DNM can be well represented using logic circuit symbols as shown in Fig. 8. Figure 8 illustrates a complete structural process of DNM training. When the input data is fed into the model as activation potentials, the final soma output is obtained through filtering, pruning, and other operations.

We pick 7 different types of classification problems from the UC Irvine machine learning repository, containing medical, biological, physical, and other fields. Table 9 summarizes the relevant properties about the data sets and the training. In this training experiment, we choose MCSMA, SMA, HHO, SSA, and a classical back-propagation algorithm(BP) as the comparison training algorithms. BP is the most popular and successful neural network learning algorithm. It utilizes two phases of forward and backward propagation to achieve a predetermined target outcome^64^. To ensure reliable and fair experimental results, the number of evaluations is 30000, the sample ratio of training samples to test samples is 1:1. Table 10 lists the results of the four compared algorithms on the training and test sets. The experimental data are measured by the overall accuracy. From the comparison results, MCSMA achieves the best accuracy on the five test sets, indicating that the proposed algorithm achieves the most correct sample classifications on multiple classification problems.

Through the above real-world problem test sets and neural network training experiments, we can conclude that MCSMA can cope well with some real-world practical optimization problems and classification problems.

**Table 9:** The relevant properties settings of data sets and the training process.

Data set	# of features	# of classes	# of training sets	# of test sets	# of dendrite (DNM)
XOR	2	2	8	8	4
IRIS	4	3	75	75	6
LIVER	6	2	173	172	8
GLASS	9	2	107	107	11
CANCER	9	2	350	349	11
WINE	13	3	89	89	14
HABERMANS	3	2	306	306	10

**Table 10:** The overall accuracy results on classification data sets.

Dataset	SMA		MCSMA		HHO		SSA		BP	
	Train(%)	Test(%)	Train(%)	Test(%)	Train(%)	Test(%)	Train(%)	Test(%)	Train(%)	Test(%)
XOR	100.000	**100.000**	100.000	**100.000**	100.000	**100.000**	100.000	**100.000**	97.500	97.500
IRIS	97.600	94.000	97.333	93.200	96.933	94.133	96.533	**94.533**	92.667	90.667
LIVER	69.360	63.815	69.942	**65.607**	63.837	59.653	59.186	57.688	59.302	56.647
GLASS	91.682	88.224	96.075	91.215	96.075	**92.243**	95.981	89.626	96.168	91.963
CANCER	97.221	96.286	96.991	**96.429**	97.994	96.371	97.249	95.457	96.934	95.771
WINE	68.202	62.022	91.461	**89.775**	87.640	83.371	91.011	87.753	72.697	65.843
HABERMANS	78.954	71.307	76.601	**74.183**	76.536	73.268	74.510	72.810	80.327	71.503

Significance values are given in Bold.

## Discussion

In this section, we perform a comprehensive discussion about the parameter and properties of MCSMA. Furthermore, an analysis regarding the effectiveness of our proposed MLE-based selection mechanism is presented, comparing it with traditional chaotic improvement methods. We hope to have a valid analysis and discussion of the underlying operational structure of the algorithm.

### Parameter discussion

In MCSMA, whether the algorithm performs a chaotic local search is determined by an evaluation value. When $rand \le \eta$, the mold individuals move forward or backward along the original trajectory; when $rand \ge \eta$, the population starts a chaotic local search and performs a chaotic oscillatory motion along the original motion trajectory. With the complementarity of such different search behaviors, the omission of the solution space is complemented and the local trap is jumped out. $\eta$ is a random value that can take the value of (0, 1], and it may have an impact on the performance of the algorithm. We could not determine the specific value of $\eta$ that would result in the maximum improvement in the ability of MCSMA, so we designed a set of controlled experiments. Ten real values of $\eta$ are taken at 0.1 intervals and tested on the CEC2017 problem set.

**Table 11:** The parameter $\eta$ control group experimental results on CEC2017.

	$\eta =0.1$	$\eta =0.2$	$\eta =0.3$	$\eta =0.4$	$\eta =0.5$	$\eta =0.6$	$\eta =0.7$	$\eta =0.8$	$\eta =0.9$	$\eta =1$
F1	7.479E+03	9.450E+03	8.513E+03	6.162E+03	7.969E+03	6.942E+03	7.263E+03	7.222E+03	7.290E+03	8.481E+03
F2	3.006E+02	3.009E+02	3.017E+02	3.016E+02	3.012E+02	3.020E+02	3.022E+02	3.026E+02	3.025E+02	3.026E+02
F3	4.958E+02	4.977E+02	4.965E+02	4.947E+02	5.021E+02	4.988E+02	4.977E+02	4.980E+02	4.953E+02	5.002E+02
F4	6.002E+02	5.745E+02	5.720E+02	5.701E+02	5.680E+02	5.778E+02	5.773E+02	5.738E+02	5.719E+02	5.708E+02
F5	6.037E+02	6.011E+02	6.031E+02	6.024E+02	6.018E+02	6.037E+02	6.041E+02	6.049E+02	6.048E+02	6.052E+02
F6	8.131E+02	8.051E+02	8.030E+02	8.032E+02	8.080E+02	8.087E+02	8.082E+02	8.055E+02	8.098E+02	8.120E+02
F7	9.124E+02	8.910E+02	8.721E+02	8.758E+02	8.850E+02	8.742E+02	8.762E+02	8.780E+02	8.724E+02	8.727E+02
F8	2.122E+03	1.273E+03	1.263E+03	1.240E+03	1.107E+03	1.190E+03	1.152E+03	1.111E+03	1.073E+03	1.192E+03
F9	3.940E+03	3.943E+03	3.980E+03	3.970E+03	3.690E+03	3.789E+03	3.859E+03	3.794E+03	4.044E+03	3.827E+03
F10	1.173E+03	1.174E+03	1.175E+03	1.175E+03	1.182E+03	1.177E+03	1.182E+03	1.187E+03	1.189E+03	1.194E+03
F11	1.157E+05	1.081E+05	1.091E+05	1.297E+05	1.125E+05	1.081E+05	1.092E+05	1.015E+05	1.018E+05	1.226E+05
F12	3.260E+04	2.427E+04	2.891E+04	2.374E+04	2.537E+04	2.388E+04	2.629E+04	2.568E+04	2.749E+04	2.595E+04
F13	1.187E+04	1.381E+04	1.377E+04	1.298E+04	1.096E+04	1.177E+04	1.281E+04	1.429E+04	1.154E+04	1.196E+04
F14	2.111E+04	1.759E+04	1.485E+04	2.067E+04	2.011E+04	1.681E+04	1.907E+04	1.688E+04	1.819E+04	2.011E+04
F15	2.266E+03	2.307E+03	2.328E+03	2.274E+03	2.290E+03	2.221E+03	2.270E+03	2.240E+03	2.266E+03	2.275E+03
F16	2.067E+03	2.022E+03	2.020E+03	2.069E+03	2.011E+03	2.017E+03	2.067E+03	2.051E+03	2.044E+03	2.061E+03
F17	2.520E+05	2.497E+05	2.606E+05	3.089E+05	2.532E+05	2.823E+05	3.281E+05	2.085E+05	2.741E+05	2.283E+05
F18	2.418E+04	2.790E+04	2.425E+04	2.290E+04	2.803E+04	2.724E+04	2.759E+04	3.369E+04	3.272E+04	2.991E+04
F19	2.442E+03	2.430E+03	2.376E+03	2.394E+03	2.429E+03	2.416E+03	2.437E+03	2.428E+03	2.395E+03	2.384E+03
F20	2.403E+03	2.374E+03	2.379E+03	2.376E+03	2.371E+03	2.370E+03	2.371E+03	2.372E+03	2.374E+03	2.381E+03
F21	5.168E+03	4.937E+03	5.110E+03	5.092E+03	4.744E+03	5.181E+03	4.840E+03	4.592E+03	3.997E+03	3.294E+03
F22	2.735E+03	2.728E+03	2.723E+03	2.718E+03	2.720E+03	2.720E+03	2.721E+03	2.727E+03	2.728E+03	2.724E+03
F23	2.903E+03	2.899E+03	2.900E+03	2.897E+03	2.897E+03	2.899E+03	2.901E+03	2.900E+03	2.896E+03	2.896E+03
F24	2.886E+03	2.888E+03	2.891E+03	2.893E+03	2.888E+03	2.891E+03	2.891E+03	2.892E+03	2.891E+03	2.891E+03
F25	4.496E+03	4.329E+03	4.329E+03	4.352E+03	4.371E+03	4.404E+03	4.374E+03	4.392E+03	4.346E+03	4.385E+03
F26	3.208E+03	3.208E+03	3.210E+03	3.208E+03	3.208E+03	3.211E+03	3.213E+03	3.209E+03	3.208E+03	3.212E+03
F27	3.219E+03	3.227E+03	3.241E+03	3.239E+03	3.227E+03	3.239E+03	3.251E+03	3.247E+03	3.247E+03	3.248E+03
F28	3.654E+03	3.628E+03	3.644E+03	3.637E+03	3.613E+03	3.626E+03	3.629E+03	3.611E+03	3.603E+03	3.627E+03
F29	1.201E+04	1.220E+04	1.285E+04	1.262E+04	1.252E+04	1.335E+04	1.329E+04	1.313E+04	1.347E+04	1.388E+04
Rank	8	4	6	3	1	2	9	7	5	10

Table 11 reveals the performance under 10 different parameters of MCSMA on CEC2017 test sets. We perform a Friedman test on ten sets of data to arrive at a final ranking. When $\eta$ takes 0.5, the algorithm has the first Friedman rank and performs the best. By analyzing the data, we can conclude that the algorithm performs relatively well when $\eta$ takes 0.4, 0.5, and 0.6. This indicates that taking values for the median allows for a good coupling of the chaos operator and the slime mold search. Therefore, we set the parameter $\eta$ of MCSMA as 0.5 in this study.

### Population movement trajectory analysis

For meta-heuristics, the algorithm maintains the population updates and motions to perform a probe in the solution space. The size and motion of the population make a direct difference in the robustness and performance of the algorithm. Individuals in the population intelligence first explore the space extensively, looking for more information to decide whether they can obtain sufficient rewards. When many individuals always choose a certain region or follow a specific trajectory, it is likely to be trapped into a local optimum. At this point, intelligent individuals are needed to develop optimal solutions or decisions around known search regions in which to help the algorithm escape the trap of local optimal. The issue of how to reasonably design the algorithm’s strategy at different stages is a critical issue.

**Figure 9:**
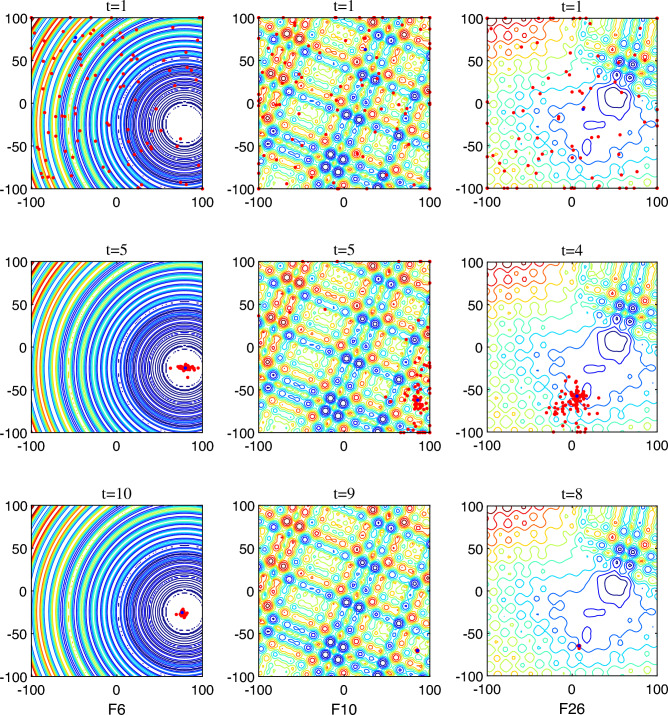
The population movement trajectory on F6, F10 and F26.

We visually observe the different stages of the algorithm by the search trajectory of the population. Figure 9 shows the trajectory and trend of the MCSMA on the three typical problems (F6, F10, and F26) of the CEC2017 test set for the slime population. On the multi-peaked function F6, we can see that the slime molds are randomly spread throughout the space at the beginning of the iteration. This is a search process, with the population searching aimlessly for information about possible food. When the number of iterations reaches 5, we can see that the mucilaginous population rapidly clusters into areas of possible optimal solutions. As the number of iterations increases, the individual slime bacteria arrange their search strategy according to the available information and implement a chaotic local search around this region. From $t = 5$ to $t = 10$, this process interprets the exploitation of a particular region by a population of slime bacteria. We can clearly see the population-specific search strategy of MCSMA in the two different types of test functions, F10 and F26. This indicates that the search strategy of MCSMA rationally designs the trajectory of the population to achieve the coordination of search and exploitation.

### Validity of MLE

When algorithms use multiple improvement mechanisms, it is difficult to determine which of these improvements on the algorithm is beneficial. Each algorithm has extremely complex mathematical workings behind it, and there is a degree of black box effect. In this section, we hope to prove that this new MLE-based probabilistic adjustment mechanism we proposed has a genuine improvement on the algorithm.

To investigate the impact of MLE of interest on the chaotic behavior perturbation of the search system, we design a group of controlled ablation experiments. In this set of experiments, we verify the effectiveness of this MLE-based roulette selection mechanism by using MCSMA and a multi-chaotic slime mold algorithm (CSMA) as control groups. CSMA also uses a success probability roulette mechanism to involve chaotic maps, but there is no quadratic weight probability adjustment based on MLE. The local search operator selects chaotic maps based solely on the quality of solutions without considering the guiding significance of the mathematical properties of chaotic maps on the selection weights. Table 12 manifests the comparative outcomes between MCSMA and CSMA on CEC2017. From Table 12, it can be observed that in the case of unimodal problems F1 and F2, the inclusion of MLE seems to have a negative effect, indicating that excessive chaotic perturbations are not beneficial for the evolutionary search of the population in extremely low-dimensional unimodal problems. However, in almost all multimodal and complex hybrid problems, MCSMA achieves optimal values and demonstrates significantly prior performance compared to CSMA without the MLE-based optimization of weights. The consequence of “*W*/*T*/*L*” shows the validity of the design of MLE-based roulette. This can prove that the proposed strategy of reasonably choosing the best chaotic local operator based on the inherent properties of chaos, i.e., MLE, is genuinely valid.

**Table 12:** The comparison experimental results of MCSMA and CSMA on CEC2017 in Dimension 30.

Algorithm	F1	F2	F3	F4
	MEAN STD	MEAN STD	MEAN STD	MEAN STD
MCSMA	9.401E+03 ± 7.717E+03	3.015E+02 ± 5.027E−01	**4.948E+02** ± **1.382E+01**	**5.714E+02** ± **2.010E+01**
CSMA	**7.765E+03** ± **7.356E+03** $\approx$	**3.001E+02** ± **1.705E-01** −	4.895E+02 ± 3.831E+00 $\approx$	5.856E+02 ± 2.239E+01 +
	F5	F6	F7	F8
	MEAN STD	MEAN STD	MEAN STD	MEAN STD
MCSMA	6.022E+02 ± 7.549E−01	**8.034E+02** ± **1.943E+01**	**8.765E+02** ± **2.086E+01**	**1.335E+03** ± **8.465E+02**
CSMA	**6.007E+02** ± **3.881E-01** −	8.187E+02 ± 1.802E+01 $\approx$	8.850E+02 ± 2.401E+01 +	1.668E+03 ± 7.245E+02 +
	F9	F10	F11	F12
	MEAN STD	MEAN STD	MEAN STD	MEAN STD
MCSMA	**3.824E+03** ± **5.848E+02**	**1.172E+03** ± **3.527E+01**	**1.249E+05** ± **7.938E+04**	**2.549E+04** ± **2.525E+04**
CSMA	4.037E+03 ± 5.684E+02 +	1.225E+03 ± 4.214E+01 +	9.906E+05 ± 8.114E+05 +	2.768E+04 ± 2.587E+04 $\approx$
	F13	F14	F15	F16
	MEAN STD	MEAN STD	MEAN STD	MEAN STD
MCSMA	**1.140E+04** ± **7.108E+03**	**1.866E+04** ± **1.559E+04**	**2.305E+03** ± **3.019E+02**	**2.045E+03** ± **1.811E+02**
CSMA	4.124E+04 ± 2.184E+04 +	2.876E+04 ± 1.394E+04 +	2.446E+03 ± 3.099E+02 +	2.156E+03 ± 1.888E+02 +
	F17	F18	F19	F20
	MEAN STD	MEAN STD	MEAN STD	MEAN STD
MCSMA	**2.319E+05** ± **1.984E+05**	**2.003E+04** ± **1.993E+04**	2.432E+03 ± 1.915E+02	**2.371E+03** ± **1.949E+01**
CSMA	2.973E+05 ± 2.510E+05 +	3.047E+04 ± 2.055E+04 +	**2.348E+03** ± **1.684E+02** −	2.394E+03 ± 2.595E+01 +
	F21	F22	F23	F24
	MEAN STD	MEAN STD	MEAN STD	MEAN STD
MCSMA	**5.088E+03** ± **1.104E+03**	**2.720E+03** ± **1.755E+01**	**2.898E+03** ± **2.207E+01**	2.888E+03 ± 1.804E+00
CSMA	5.466E+03 ± 8.774E+02 +	2.738E+03 ± 1.910E+01 $\approx$	2.926E+03 ± 2.755E+01 +	**2.887E+03** ± **1.779E+00** $\approx$
	F25	F26	F27	F28
	MEAN STD	MEAN STD	MEAN STD	MEAN STD
MCSMA	**4.350E+03** ± **3.559E+02**	**3.212E+03** ± **1.068E+01**	**3.237E+03** ± **2.829E+01**	**3.590E+03** ± **1.439E+02**
CSMA	4.654E+03 ± 2.382E+02 +	3.212E+03 ± 1.162E+01 $\approx$	3.242E+03 ± 3.660E+01 $\approx$	3.707E+03 ± 1.937E+02 +
	F29	*W*/*T*/*L*		
	MEAN STD			
MCSMA	**1.148E+04** ± **4.274E+03**	${-}{-}/{-}{-}/{-}{-}$		
CSMA	1.577E+04 ± 4.348E+03 +	17/8/4		

Significance values are given in Bold.

### Analysis of time complexity

The evaluation of algorithmic time complexity aims to estimate how the execution time and resource usage of a program increase with the growth of input size. In initial setting, *N* denotes the population size; *D* is the dimension scale; *T* represents the number of iterations. The time complexity of MCSMA can be calculated by follows:

1. The process of initializing population needs $O(N\times D)+O(N)$.
2. Generating correlation coefficient matrix *C* needs *O*(*N*).
3. The fitness evaluation and sorting of individuals costs $O(N\times T\times (1+log N))$.
4. Updating the $M_{i}$ requires $O(N\times T\times D)$.
5. The phase of chaotic local search costs $O(N\times T\times D)$.

So the time complexity of MCSMA can be summarized as $O((N+2N\times T)\times D+2N+N\times T\times (1+logN))$. According to^27^, the original SMA’s time complexity is $O((1+N\times T)\times D+N\times T\times (1+logN))$. It can be observed that the time complexity of both algorithms remain at the linear-logarithmic order, indicating that our proposed improvement method does not lead a significant increase in program complexity and does not require sacrificing computational resources for performance improvements.

## Conclusion

In this paper, we propose a novel algorithm, MCSMA, that incorporates a multi-chaotic local operator while retaining the unique sticky bacteria feedback search in SMA. We consider for the first time the fundamental property of chaotic motion, i.e., the maximum Lyapunov exponent, and add it as an evaluation criterion to the meritocratic multi-chaotic roulette wheel. By constructing the MLE correlation matrix of the chaotic map as a moderating factor for probabilistic adjustment, the most efficient and suitable chaotic operator for the algorithm is screened. We compare the performance of MCSMA on three different types of test sets, i.e., IEEE CEC2017, CEC2011, and a challenging neural network learning task. Experimental results verify the effectiveness and feasibility of MCSMA.

It has been a pressing challenge for computational intelligence to effectively address the need of compensating for some specific drawbacks of algorithms while avoiding compromising their advantages^12,65^. We aspire to construct a general enhancement mechanism to improve the local exploitation capability of existing algorithms, and the issue of how to optimize and generalize the structure of such chaotic local operators is a focus of future work. It is also worthwhile to apply MCSMA to other areas, such as control scheduling, industrial modeling, and data processing^66,67^.

## Data Availability

The data that support the findings of this study are available from the corresponding author upon reasonable request.
